# The Role of OmpR in the Expression of Genes of the KdgR Regulon Involved in the Uptake and Depolymerization of Oligogalacturonides in *Yersinia enterocolitica*

**DOI:** 10.3389/fcimb.2017.00366

**Published:** 2017-08-15

**Authors:** Marta Nieckarz, Adrianna Raczkowska, Karolina Jaworska, Ewa Stefańska, Karolina Skorek, Dorota Stosio, Katarzyna Brzostek

**Affiliations:** Department of Applied Microbiology, Faculty of Biology, Institute of Microbiology, University of Warsaw Warsaw, Poland

**Keywords:** OmpR, KdgR, KdgM porins, *Yersinia enterocolitica*, pectate lyase

## Abstract

Oligogalacturonide (OGA)-specific porins of the KdgM family have previously been identified and characterized in enterobacterial plant pathogens. We found that deletion of the gene encoding response regulator OmpR causes the porin KdgM2 to become one of the most abundant proteins in the outer membrane of the human enteropathogen *Yersinia enterocolitica*. Reporter gene fusion and real-time PCR analysis confirmed that the expression of *kdgM2* is repressed by OmpR. We also found that *kdgM2* expression is subject to negative regulation by KdgR, a specific repressor of genes involved in the uptake and metabolism of pectin derivatives in plant pathogens. The additive effect of *kdgR* and *ompR* mutations suggested that KdgR and OmpR regulate *kdgM2* expression independently. We confirmed that *kdgM2* occurs in an operon with the *pelP* gene, encoding the periplasmic pectate lyase PelP. A pectinolytic assay showed strong upregulation of PelP production/activity in a *Y. enterocolitica* strain lacking OmpR and KdgR, which corroborates the repression exerted by these regulators on *kdgM2*. In addition, our data showed that OmpR is responsible for up regulation of the *kdgM1* gene encoding the second specific oligogalacturonide porin KdgM1. This indicates the involvement of OmpR in the reciprocal regulation of both KdgM1 and KdgM2. Moreover, we demonstrated the negative impact of OmpR on *kdgR* transcription, which might positively affect the expression of genes of the KdgR regulon. Binding of OmpR to the promoter regions of the *kdgM2-pelP-sghX* operon, and *kdgM1* and *kdgR* genes was confirmed using the electrophoretic mobility shift assay, suggesting that OmpR can directly regulate their transcription. We also found that the overexpression of porin KdgM2 increases outer membrane permeability. Thus, OmpR-mediated regulation of the KdgM porins may contribute to the fitness of *Y. enterocolitica* in particular local environments.

## Introduction

*Yersinia enterocolitica*, an enteropathogenic bacterium of the genus *Yersinia* in the Enterobacteariaceae family is found in various ecological niches associated with the human body and free-living in the environment (Bottone, 1997). To reside in these greatly different habitats, *Y. enterocolitica* requires the ability to rapidly adapt to fluctuations in various physico-chemical factors (Straley and Perry, 1995). Adaptation to new growth conditions involves the reorganization of gene expression, which is mediated partly by two-component regulatory systems (TCSs) (Stock et al., 1989; Hoch and Silhavy, 1995). TCSs play a role in diverse signaling processes and are widespread in bacteria, including the genus *Yersinia* (Marceau, 2005; Flamez et al., 2008; O'Loughlin et al., 2010). The EnvZ/OmpR regulatory system of non-pathogenic *Escherichia coli* K-12 is the most well characterized TCS, consisting of the transmembrane histidine kinase EnvZ and response regulator OmpR, which acts to modulate gene transcription (Kenney, 2002).

Much of our knowledge of the regulatory activities of EnvZ/OmpR has been derived from studies on the regulation of the outer membrane (OM) OmpC and OmpF porins in response to changes in the osmolarity of the environment (Russo and Silhavy, 1991). OmpC and OmpF are general porins that facilitate the passive and non-specific diffusion of low molecular weight hydrophilic substances across the OM (Nikaido, 2003). OmpR is a transcription factor that plays a role in both the positive and negative regulation of the *ompC* and *ompF* genes. Besides these general porin genes, other targets of OmpR have been identified in *E. coli* and other enterobacteria. OmpR is involved in the regulation of flagella synthesis (Shin and Park, 1995), fatty acid transport (Higashitani et al., 1993), the stationary-phase acid tolerance reponse (Bang et al., 2000) and curli fiber formation (Jubelin et al., 2005). Moreover, a role for OmpR in controlling the virulence properties of pathogenic bacteria has been demonstrated (Bernardini et al., 1990; Lee et al., 2000; Hu et al., 2009; Cameron and Dorman, 2012). The function of OmpR is important in the physiology and virulence of *Y. enterocolitica* (Dorrell et al., 1998; Brzostek et al., 2003; Raczkowska et al., 2011; Skorek et al., 2013).

Outer membrane proteins (OMPs) play a crucial role in the adaptation of bacterial cells to changes in the environment (Nikaido, 2003). Changes in the OMP composition allow bacteria to adapt to diverse environments, are associated with drug resistance, and are involved in bacterial pathogenesis. Recently, a proteomic approach was used to investigate OmpR-dependent OMP expression in *Y. enterocolitica* (Nieckarz et al., 2016). Comparative LC-MS/MS analysis identified a large panel of proteins whose expression is negatively or positively regulated by OmpR. Among the OMPs that are negatively affected by OmpR, one displayed high similarity to the KdgM proteins of *Dickeya dadantii* (formerly *Erwinia chrysanthemi*), a phytopathogenic member of the Enterobacteriaceae (Hugouvieux-Cotte-Pattat et al., 1996; Blot et al., 2002; Condemine and Ghazi, 2007). Two proteins of the *D. dadantii* KdgM family, namely KdgM and KdgN, are well characterized OM porins involved in the import of long oligogalacturonides (OGAs), the products of pectin degradation (Blot et al., 2002; Condemine and Ghazi, 2007). The synthesis of these specific porins is strongly induced by the presence of pectic derivatives and is controlled by KdgR, a general repressor of operons/genes involved in pectin catabolism in *D. dadantii* (Nasser et al., 1992; Rodionov et al., 2004). The KdgR regulon of *D. dadantii* includes genes encoding secreted pectinases as well as periplasmic and cytoplasmic enzymes that participate in the depolymerization of pectin derivatives. The end product of the pectin degradation pathway is 2-keto-3-deoxygluconate (KDG), which is used as a source of carbon and energy, and as a direct effector of KdgR activity. The interaction of KdgR with KDG releases this transcriptional regulator from operators located in the regulatory regions of target genes, thus derepressing their expression (Reverchon et al., 1991; Nasser et al., 1994).

Comparative genomic analysis revealed the presence of an incomplete pectin degradation pathway in a variety of enterobacteria, including pathogenic *Yersiniae*, i.e., enteropathogenic *Y. enterocolitica* and the plague bacillus *Y. pestis* (Rodionov et al., 2004). Pathogenic *Yersiniae* lack extracellular pectinases as well as the Out transport system necessary for their secretion. Furthermore, other enzymes that are responsible for the degradation of pectin derivatives are missing. In comparison with the large number of such enzymes in *D. dadantii*, only three pectate lyases have been detected in *Y. enterocolitica* (Abbott and Boraston, 2008; Hugouvieux-Cotte-Pattat et al., 2012).

In this study we have used available genomic data and bioinformatics tools to identify an incomplete pectin degradation pathway in *Y. enterocolitica* subsp. *palearctica*, which includes two porins of the KdgM family (KdgM1 and KdgM2). The role of *Y. enterocolitica* OmpR in the modulation of *kdgM* genes expression was characterized and this regulatory activity was correlated with variations in selected physiological properties of this enteropathogen that may be important for stress resistance.

## Materials and methods

### Bacterial strains and growth conditions

The bacterial strains used in this study are described in Table S1. Unless indicated, *Y. enterocolitica* strains were cultured at 26°C in LB medium (10 g tryptone, 5 g yeast extract and 5 g NaCl per liter) or in Minimal medium A (MMA; Miller, 1992). *E. coli* strains were grown at 37°C in LB medium. *Rhizobium etli* CE3 was grown at 26°C in TY medium (Beringer, 1974). As required, media were supplemented with glycerol (Gl; 0.2% w/v), polygalacturonic acid (PGA; Sigma-Aldrich) that had been hydrolyzed by autoclaving to produce a mixture of oligogalacturonides (OGAs; 0.4% w/v) (Goubet et al., 2003) or pectin (from citrus peel, 0.2% w/v; Sigma-Aldrich). L-(+)-arabinose was added to growth medium at a concentration of 0.2% (w/v) to induce expression from the arabinose-regulated promoter in pBAD18Km. Antibiotics were used for selection at the following concentrations: nalidixic acid (Nal)−30 μg/ml, chloramphenicol (Cm)−25 μg/ml, kanamycin (Km)−50 μg/ml, gentamicin (Gm)−40 μg/ml, tetracycline (Tet)−12.5 μg/ml, trimethoprim (Tp)−50 μg/ml. To test the effect of high osmolarity, exponential phase bacterial cultures (OD_600_ ~ 0.4-0.5) were grown at 26°C (with shaking 150 rpm) for 2 h in Nutrient Broth medium (3 g beef extract, 5 g peptone per liter) containing 0 mM, 100 mM or 350 mM NaCl.

### Molecular biology techniques

All DNA manipulations, including polymerase chain reaction (PCR), restriction digests, ligations and DNA electrophoresis, were performed as previously described (Sambrook and Russell, 2001). The PCR was routinely performed using Taq DNA polymerase or, when fragments were used for cloning, Phusion High-Fidelity DNA Polymerase (Thermo Scientific). Oligonucleotide primers used for PCR and sequencing were purchased from Genomed S.A. (Warsaw, Poland) and are listed in Table S2. Plasmids used in this study are described in Table S1. DNA sequencing was performed by Genomed S.A.

### Isolation of outer membrane proteins and SDS-PAGE analysis

*Y. enterocolitica* strains were grown overnight at 26°C with shaking (150 rpm) in LB medium without or with OGAs. Outer membrane proteins isolation and SDS-PAGE analysis were performed as described in Nieckarz et al. (2016). The final protein concentrations in the samples were estimated using the RC-DC protein assay (Bio-Rad) and normalized by dilution in Laemmli buffer (Sambrook and Russell, 2001). The OM samples were mixed with sample buffer and the OMPs extracted by boiling for 10 min. Samples (50 μg of protein) were separated by electrophoresis on 10 or 12% SDS-polyacrylamide gels (SDS-PAGE) and individual polypeptide bands were visualized by Coomassie blue staining. Densitometry of stained gels was performed using Bio-Rad Image Lab software.

### Construction of a *kdgM2′-′rfp* translational fusion plasmid

To examine the regulation of KdgM2 expression, a translational *kdgM2* fusion to *rfp* (red fluorescent protein gene) was constructed in plasmid pBBR1MCS-5 (Gm^R^). An 1,125-bp DNA fragment comprised of a sequence extending from 264 bp upstream of the *kdgM2* start codon (with *kdgM2* native ribosome binding site) plus the first 10 codons of the open reading frame (ORF) fused to an 815-bp *rfp* ORF was synthesized (GeneCust, Luxembourg). This synthetic *kdgM2*′-′*rfp* fragment had a BamHI site at the 5′ end and an EcoRI site at the 3′ end, and these restriction endonucleases were used to clone it using the same restriction sites in vector pBluescript II SK(+) (Agilent Technologies). Plasmid DNA of the recombinant construct was digested with BamHI and EcoRI to release a 1125-bp fragment and this was then cloned into vector pBBR1MCS-5 using the same sites to give plasmid pBKRFP. This construct was introduced into *E. coli* S17-1 λ*pir* and then transferred by conjugation into *Y. enterocolitica* strain Ye9N and strain AR4, the *ompR* deletion mutant (Δ*ompR*::Km). Transconjugants of Ye9N and AR4 with pBKRFP were selected on LB agar plates containing Gm and Nal and Gm and Km, respectively. To confirm the sequence of the *kdgM2*′-′*rfp* fusion a PCR amplicon generated with primers LL1 and RR4 (Table S2) was sequenced. For experiments with *Y. enterocolitica* strains carrying the Δ*kdgR*::Gm mutation, the *kdgM2*′-′*rfp* fusion was also expressed from plasmid pBKRFP-Tp, a derivative of pBKRFP containing a trimethoprim (Tp) resistance cassette. To construct pBKRFP-Tp, the Tp cassette from plasmid p34E-Tp on a SalI fragment was inserted into the unique SalI site of pBKRFP. Plasmid pBKRFP-Tp was transferred to the *Y. enterocolitica kdgR* mutant (strain ES1) and the *ompRkdgR* mutant (strain AR11) by triparental mating with *E. coli* TG1 (pBKRFP-Tp) as the donor and *E. coli* DH5α/pRK2013 as the helper. Transconjugants of ES1 and AR11 with pBKRFP-Tp were selected on LB agar plates containing Nal and Tp.

### Measurement of RFP fluorescence

Two hundred microliter of culture of each strain were transferred to wells of 96-well black flat-bottomed microtitre plates with a clear base (Greiner Bio-One). Absorbance at 600 nm and RFP fluorescence (excitation 555 nm; emission 632 nm) were measured using a TECAN Infinite M200PRO microplate reader. Specific RFP fluorescence was expressed as the relative fluorescence intensity (RFU) divided by the OD_600_ after subtracting the values of a blank sample. Each culture was assayed in triplicate and the reported values are the means from three independent cultures. To test the effect of high osmolarity, bacterial cultures were grown to exponential phase in NB medium containing 350 mM NaCl. Then, 200 μl of the treated or control cell suspensions were transferred to 96-well plates and OD_600_ and RFP fluorescence were measured as before.

### Construction of *kdgR* and *kdgM2* deletion mutants

The Δ*kdgR*::Gm and Δ*kdgM2*::Gm deletion mutants of *Y. enterocolitica* Ye9N and the *ompR* mutant AR4 were constructed by homologous recombination using suicide vector pDS132 (Philippe et al., 2004). Constructs were prepared containing overlap extension PCR products to mutate *kdgR* and *kdgM2* by insertion of a Gm^R^ cassette via allelic exchange at the native chromosomal loci of *Y. enterocolitica*. For each gene, three DNA fragments were PCR-amplified using primers listed in Table S2, with *Y. enterocolitica* chromosomal DNA (for flanking regions) or plasmid pBBR1MCS-5 Gm^R^ (for the Gm^R^ cassette) as the templates. The following primer pairs were used for the construction of *kdgR* mutants: KdgR1/KdgR2 generated fragment A-a 705-bp sequence upstream of the *kdgR* ORF; KdgR3/KdgR4 generated fragment B-an 802-bp Gm^R^ cassette; KdgR5/KdgR6 generated fragment C-a 684-bp sequence downstream of the *kdgR* gene. The following primer pairs were used for the construction of *kdgM2* mutants: KdgM1/KdgM2 generated fragment A-a sequence comprising 437 bp upstream of the *kdgM2* gene plus the first 250 bp of the ORF; KdgM3/KdgM4 generated fragment B-an 802-bp Gm^R^ cassette; KdgM5/KdgM6 generated fragment C-a sequence comprising the last 74 bp of the *kdgM2* ORF plus 623 bp downstream of this ORF. Products A, B and C were used as the template with flanking primers KdgR1 and KdgR6 (*kdgR* mutagenesis) or KdgM1 and KdgM6 (*kdgM2* mutagenesis) to generate the final PCR products. These amplicons were purified, digested with XbaI and then individually cloned into the corresponding restriction site in suicide vector pDS132, yielding constructs pDSkdgR and pDSkdgM2, respectively. These plasmids were introduced into *E. coli* S17-1 λ*pir* by transformation, with selection on chloramphenicol and gentamicin, and then sequenced to confirm the absence of errors. Finally, pDSkdgR and pDSkdgM2 were introduced into *Y. enterocolitica* strains Ye9N and AR4 by biparental mating. Transconjugants containing single crossovers of the allelic exchange plasmid integrated into the Ye9N or AR4 genomes were selected in LB supplemented with chloramphenicol, gentamicin plus nalidixic acid (Ye9N) or kanamycin (for AR4). Integration after a single crossover was verified by PCR. To force the second recombination, the single-crossover strains were plated on LB containing gentamicin and 10% (w/v) sucrose, and incubated at room temperature for 48 h. Sucrose-resistant colonies were screened for the loss of chloramphenicol resistance (encoded by the vector). The correct allelic exchange was verified for the *kdgR* and *kdgM2* mutants by PCR using the primer pairs KdgR0/KdgR7 and KdgM0/KdgM7, respectively. Sequencing of the amplified fragments confirmed that the mutagenesis was correct. The Δ*kdgR*::Gm mutants in Ye9N and AR4 were named ES1 and AR11, respectively. The Δ*kdgM2*::Gm mutants in these strains were named MN1 and AR10, respectively.

### Construction of *kdgR*::*lacZ, pehX::lacZ* and *pelW-togMNAB::lacZ* transcriptional fusion plasmids

To obtain *kdgR*::*lacZ, pehX::lacZ* and *pelW-togMNAB::lacZ* transcriptional fusions, DNA fragments containing the promoters of the *kdgR* and *pehX* genes and the *pelW-togMNAB* operon were amplified from *Y. enterocolitica* chromosomal DNA by PCR using the primer pairs KdgREcoRI/KdgRKpnI, PehXEcoRI/PehXKpnI and PelWEcoRI/PelWKpnI, respectively. The amplified fragments were digested with EcoRI/KpnI and cloned into the corresponding sites of reporter vector pCM132Gm [derivative of plasmid pCM132 (Marx and Lidstrom, 2001) containing a gentamicin resistance cassette; a kind gift from Dr J. Czarnecki] upstream of a promoterless *lacZ* gene. The resulting constructs were verified by PCR using the primer pair pCM132GmSPR1/pCM132GmSPR2 (flanking the EcoRI and KpnI recognition sequences) followed by sequencing of the amplicons. The constructs pCM132Gm-*kdgR::lacZ*, pCM132Gm-*pehX::lacZ* and pCM132Gm-*pelW-togMNAB::lacZ* were introduced into *E. coli* S17-1 λ*pir* and transferred by conjugation into *Y. enterocolitica* Ye9N and the *ompR* mutant AR4, selecting transconjugants on LB plates containing Gm and Nal or Gm and Km, respectively. The presence of these constructs in these *Y*. *enterocolitica* strains was confirmed by plasmid isolation and PCR with the primer pair pCM132GmSPR1/pCM132GmSPR2.

### Construction of *Y. enterocolitica* reporter strains carrying a chromosomal *kdgM1-lacZYA′* reporter fusion

To construct a *kdgM1* promoter*-lacZYA*′ fusion, a 565-bp fragment containing the *kdgM1* regulatory region was PCR-amplified from Ye9 chromosomal DNA using primer pair KdgM1X/KdgM1S (Table S2). The amplicon was initially cloned into the vector pDrive (Qiagen), and then the insert released by digestion with XbaI/SmaI was subcloned into suicide plasmid pFUSE cleaved with the same enzymes to place it immediately upstream of a promoterless *lacZYA*′ operon (Baumler et al., 1996). The suicide vector construct containing the *kdgM1* fragment, verified by restriction digestion and DNA sequencing, was named pFkdgM1. This plasmid was used to transform *E. coli* S17-1 λ*pir* and then introduced into *Y. enterocolitica* Ye9N, the *ompR* mutant AR4, the *kdgR* mutant ES1 and the *ompRkdgR* mutant AR11 by biparental mating. Conjugation between the donor and recipient strains was performed on LB agar plates for 18 h at room temperature. Transconjugants were then selected on LB agar plates containing antibiotics: chloramphenicol and nalidixic acid for Ye9N, chloramphenicol and kanamycin for AR4, and chloramphenicol and gentamicin for ES1 and AR11. Single-crossover homologous recombination yielded a genomic transcriptional fusion between the *kdgM1* promoter and the promoterless *lacZYA*′ operon. The correct insertion of the suicide vector was verified by PCR using one primer located upstream of the homologous region used for recombination (LPkdgM2683) and another primer within the *lacZ* gene (lacZH991) (Table S2), followed by sequencing of the amplicons. Strains carrying the desired transcriptional fusions were named Ye9NK1, AR4K1, ES1K1 and AR11K1.

### Construction of plasmids pkdgR-Cm and pkdgR-tet for complementation

To complement the *kdgR* mutation, the *kdgR* gene was cloned in plasmid expression vectors. The gene with its native ribosome binding site (rbs) was PCR-amplified from Ye9 chromosomal DNA using primer pairs KdgRorfBamHI/KdgRorfHindIII or KdgRorfKpnI/KdgRorfSacI (Table S2). The BamHI/HindIII *kdgR* fragment was cloned under the control of the Plac promoter in vector pHSG575 (Takeshita et al., 1987), generating plasmid pkdgR-Cm. The KpnI/SacI *kdgR* fragment was cloned under the control of the Plac promoter in vector pBBR1MCS-3 (Kovach et al., 1995) generating plasmid pkdgR-Tet. The resulting constructs were verified by DNA sequencing. Plasmid pkdgR-Cm was used to transform the *kdgR* mutant (ES1) and *ompRkdgR* mutant (AR11), both carrying the plasmid pBKRFP-Tp (expressing a *kdgM2* ′-′*rfp* fusion), by electroporation and Cm^R^Tp^R^ transformants were selected. In the same way, plasmid pkdgR-Tet was introduced into the *kdgR* mutant (ES1K1) and *ompRkdgR* mutant (AR11K1), both carrying a *kdgM1-lacZYA*′ chromosomal transcriptional fusion, and Tet^R^ Cm^R^ transformants were selected.

### Construction of a plasmid for overexpression of KdgM2

For the overproduction of KdgM2, the *kdgM2* gene was cloned under the control of the *PBAD* promoter in vector pBAD18Km. The gene with its native rbs was PCR-amplified from Ye9 chromosomal DNA using primers ARAkdgM2SacI and ARAkdgM2SphI (Table S2). The SacI/SphI *kdgM2* fragment was cloned in pBAD18Km, resulting in plasmid pBAD-KdgM2, which was verified by restriction digestion and DNA sequencing. Construct pBAD-KdgM2 was introduced into the wild-type strain Ye9 by electroporation and Km^R^ transformants were selected. To induce KdgM2 synthesis, arabinose (0.2% w/v) was added to exponential-phase cultures in liquid medium and these were examined after an additional 1 h of growth.

### RT-qPCR analysis

*Y. enterocolitica* Ye9 and the *ompR* mutant strains were grown overnight in LB medium+OGAs at 26°C. Approximately 10^9^ bacterial cells were then harvested from each culture and total RNA was isolated using a High Pure RNA Isolation Kit (Roche). After DNase treatment of the isolated RNA, cDNA was synthesized using a NG dART RT kit (Eurx). Real-Time PCR analysis was performed using a LightCycler 480 II (Roche Applied Science) with a SensiFAST SYBR No-ROX Kit (Bioline). Primers were designed by Amplicon sp. z o. o. and they are listed in Table S2. Relative quantification of gene transcription was performed using the LightCycler 480 Software 1.5.1. The data were subjected to statistical analysis using Project R (version 3.2.2.) data analysis software. The 16S rRNA gene was used as an internal reference to normalize the relative amount of target cDNA.

### Electrophoretic mobility shift assay (EMSA)

OmpR-His_6_ was expressed and purified as described previously (Nieckarz et al., 2016). The *in vitro* interaction between phosphorylated OmpR (OmpR-P) and the promoters of selected genes was examined using the EMSA, essentially as described previously (Nieckarz et al., 2016). The primers listed in Table S2 were used in PCRs with *Y. enterocolitica* genomic DNA to amplify fragments comprising the regulatory regions of the genes *kdgM1, kdgM2* and *kdgR*. To confirm binding specificity, a 304-bp fragment of the *Y. enterocolitica* Ye9 16S rRNA gene generated by PCR using primer pair 16SR1/16SR304 (Table S2) was included in all binding reactions. Ethidium bromide was used to stain DNA bands in the gels, which were visualized on a UV transilluminator.

### Pectinolytic enzyme assay

A plate assay was used to detect pectate lyase (Pel) activity in periplasmic fluid obtained using a modified osmotic shock protocol (Neu and Heppel, 1965). Pel assay medium contained 0.8% (w/v) agarose, 1% (w/v) PGA, 1% (w/v) yeast extract, 0.38 μM CaCl_2_ and 100 mM Tris-HCl, pH 8.0 (Lee et al., 2013). Wells were made in each plate using a cut pipette tip and the bottom of each well was sealed with molten 0.8% (w/v) agarose. Exponential phase cultures grown in LB medium at 26°C were adjusted to the same OD_600_, then 1 ml of each was centrifuged (4,000 × *g*, 15 min, 4°C). The cell pellets were resuspended in 0.5 ml of buffer containing 20% (w/v) sucrose, 1 mM EDTA, 30 mM Tris-HCl, pH 8.0 and incubated for 10 min at room temperature with gentle shaking. After centrifugation (13,000 × *g*, 10 min, 4°C), the cell pellets were resuspended in 0.5 ml of ice-cold pure water and incubated for 10 min at 4°C with gentle shaking. After centrifugation as before, 100 μl of the supernatants containing periplasmic fluid released by osmotic shock were added to wells of the Pel enzyme assay plates. Following incubation at 26°C for 48 h, 4 M HCl was poured onto the plates and the halo areas were measured.

### β-galactosidase assays

β-galactosidase assays were performed essentially as described by Thibodeau et al. (2004), using 96-well microtiter plates (Nest Sc. Biotech.) and a Sunrise plate reader (Tecan). The β-galactosidase activity was expressed in Miller units calculated as described previously (Thibodeau et al., 2004). Each assay was performed at least in triplicate.

### Semi-quantitative reverse transcription RT-PCR gene expression analysis

Cultures of *Y. enterocolitica* Ye9 were grown overnight in LB medium at 26°C and then total RNA was isolated from 10^7^ cells using a GeneMatrix Universal RNA Purification Kit (EURx). Following treatment with RNase-free DNase I (Sigma-Aldrich), the RNA was reverse-transcribed using AMV reverse transcriptase (Sigma-Aldrich) primed with random hexamers. The cDNA was used as the template in PCRs (RNA as a negative control) with primer pairs RTkdgMpelP1/RTkdgMpelP2 or RTpelPsghX1/RTpelPsghX2 (Table S2), specific for the *kdgM2-pelP* mRNA or the *pelP-sghX* mRNA, respectively. The amplified fragments were resolved by electrophoresis on 2% (w/v) agarose gels and visualized by staining with ethidium bromide.

### Preparation of short OGAs by polygalacturonase digestion of PGA

Short oligogalacturonides (sOGAs) were obtained using the protocol of Bellincampi et al. (1993). Briefly, 1 g of unmethylated polygalacturonic acid (PGA) was solubilized in 50 ml of 50 mM sodium acetate (pH 5.0). The solution was digested for 180 min with 0.03 mU/mg of *Aspergillus niger* polygalacturonase (Sigma-Aldrich). After heat inactivation of the enzyme, the reaction mixture was diluted with 50 mM sodium acetate to a concentration of 0.5% (w/v) PGA. Next, the digested PGA was precipitated with ethanol, incubating overnight at 4°C with shaking. The pellet was recovered by centrifugation (35,000 × *g*, 0.5 h, 4°C) and dissolved in 100 μl ultrapure water. The obtained sOGAs were analyzed by non-denaturing polyacrylamide gel electrophoresis and stained with ruthenium red (0.02%, w/v), as described previously (Potiggia et al., 2015).

### Detection of reactive oxygen species (ROS)

Intracellular production of ROS was measured using 2′,7′-dichlorofluorescein diacetate (H2DCF-DA, Molecular Probes), essentially as described by Dong et al. (2015). Exponentially growing cultures of *Y. enterocolitica* Ye9 in LB medium and *Rhizobium etli* CE3 in TY medium (OD_600_ ~0.3) were incubated with 10 μM H2DCF-DA for 30 min. This nonpolar compound passively diffuses into cells where it is converted to H2DCF by endogenous esterases and then rapidly oxidized to highly fluorescent DCF by intracellular peroxides. Excess dye was removed by extensive washing of the cells with fresh culture medium. The bacteria were treated with sOGAs obtained by polygalacturonase digestion of PGA (50 μg/ml), polymyxin B (25 μg/ml) or cell culture medium only (control) for 20 min. Fluorescence was measured using a TECAN Infinite M200PRO microplate reader: excitation 495 nm; emission 520 nm.

### Plant tissue maceration assay

The plant tissue maceration assay was performed as described by Expert and Toussaint (1985). Chicory leaves were placed in sterile Petri dishes on filter paper previously moistened with sterile water. Overnight bacterial cultures of *Y. enterocolitica* strains, *E. coli* W (ATTC 9637 strain) and *Pectobacterium carotovorum* subsp. *carotovorum* (PCM 2056 strain) grown in LB medium were diluted to an OD_600_ of 0.3. Next, 10 μl of the bacterial suspensions (about 10^8^ cells) were injected into the chicory leaves cut with a sterile scalpel. The plates were closed to maintain high humidity and incubated at 26°C. Leaf tissue maceration at the sites of inoculation was scored after 2–5 days.

### Antimicrobial susceptibility testing

The antibiotic sensitivity of *Y. enterocolitica* strains was tested using a broth micro-dilution assay. Broth microdilution was performed in sterile transparent 96-well flat-bottomed microtiter plates (Nest Sc. Biotech.). Antibiotic solutions were serially diluted 2-fold in 100 μl of Mueller-Hinton broth (MHB) in 96-well plates to produce the appropriate concentration ranges. Overnight cultures of the *Y. enterocolitica* strains were diluted to 10^5^ cfu/ml and 100 μl aliquots were added to wells of the plates containing the antibiotic dilution series. The plates were then incubated with shaking (150 rpm) at 26°C for 24 h. The OD_600_ was measured using a TECAN Infinite Pro M200PRO microplate reader. The following antibiotics were assayed: ampicillin-0.2 to 400 μg/ml; cefalotin-0.98 to 500 μg/ml; cefotaxime-0.06 to 32 μg/ml; ceftazidime-0.03 to 16 μg/ml; cephaloridine-0.98 to 500 μg/ml; chloramphenicol-0.05 to 25 μg/ml; tetracycline-0.02 to 10 μg/ml. To test the effect of hydrophobic trimethoprim (400 to 0.78 μg/ml) and gentamicin (400 to 0.78 μg/ml) strains were grown in MHB at 26°C, overnight. Next, to induce KdgM2 synthesis, arabinose (0.2% w/v) was added and these cultures were incubated an additional 1 h of growth, then diluted to 10^5^ cfu/ml and 100 μl aliquots were incubated with the antibiotic dilution series parallel at 26°C and 37°C for 24 h. The minimal inhibitory concentration (MIC) was the lowest concentration of the antimicrobial agent that prevented bacterial growth.

### Detergent sensitivity assay

The MICs of detergents were determined using a liquid culture assay as previously described (Zou et al., 2011). Briefly, overnight cultures of wild-type and the mutant strains were diluted 1:1,000 in LB medium containing 2-fold serial dilutions of the applied detergent, ranging from 800 μg/ml to 1.5625 g/ml for hexadecyltrimethylammonium bromide (CTAB) and sodium dodecyl sulfate (SDS). Growth was assessed after incubation at 26°C and 37°C with shaking (150 rpm) for 18 h. To induce KdgM2 synthesis, arabinose (0.2% w/v) was added to the cultures before their incubation for 18 h. The MIC of CTAB and SDS was the lowest concentration at which growth was completely inhibited. These assays were performed two times with identical results.

### 1-*N*-phenylnaphthylamine (NPN) accumulation assay to examine outer membrane permeability

Permeability of the *Y. enterocolotica* outer membrane was determined using the NPN uptake assay as previously described (Loh et al., 1984; Zou et al., 2011). Cultures of the test strains were grown in LB medium at 26°C or 37°C to early stationary phase. To induce KdgM2 synthesis, arabinose (0.2% w/v) was added and these cultures were incubated an additional 1 h of growth. The cells were centrifuged (8,000 × g for 1 min), and washed twice in the assay buffer (5 mM HEPES pH 7.2, 137 mM NaCl). The cells were then resuspended in the same buffer and the OD_600_ was adjusted to 1.0. 100 μl aliquots of these cell suspensions were placed in triplicate into the wells of a black 96-well fluorescence microplate (Greiner Bio-One). NPN, dissolved in acetone and then diluted in the assay buffer, was added to appropriate wells of the microplate to give a bacterial OD_600_ of 0.5 and a final NPN concentration of 10 μM. Controls containing only buffer plus NPN were included. Changes in fluorescence were then recorded using a Tecan Infinite M200PRO microplate reader: excitation 355 nm; emission 402 nm. Readings were taken every 45 s for 19.5 min. Analysis of the fluorescence values was performed using Prism 7 software (v. 7.02, GraphPad). Background fluorescence (NPN in buffer only) was subtracted from the raw values, and these results were divided by the corresponding OD_600_ values. The fluorescence of the wild-type strain at time zero was defined as 100% and all other values were normalized accordingly.

### Statistical analyses

Statistical analyses were performed using Prism 7 software (v. 7.02, GraphPad). One-way ANOVA and Tukey's multiple comparison test was used to determine statistically significant differences. In addition, the statistical significance were tested using Student's *t*-test.

## Results

### Genomic organization of the *kdgM1, kdgM2*, and *kdgR* loci of *Y. enterocolitica*

**Figure 1:**
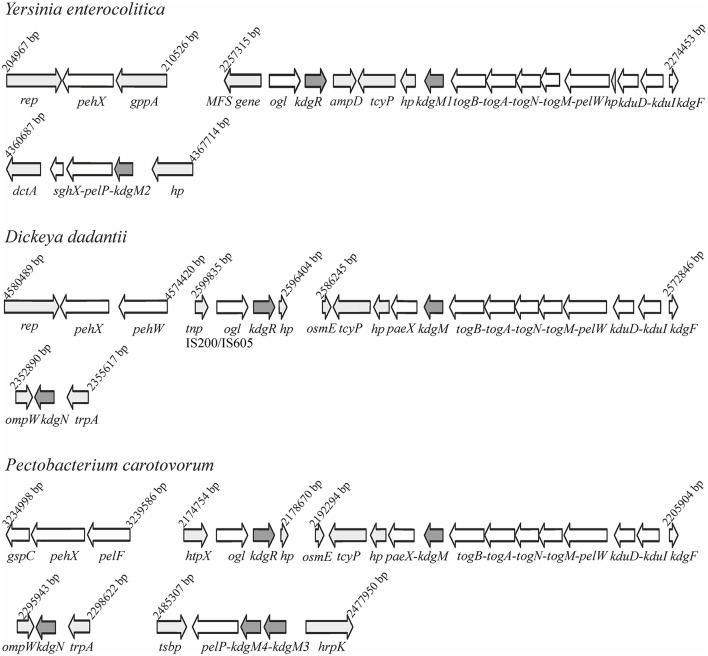
Comparison of the genomic organization of the *kdgM1, kdgM2*, and *kdgR* loci in *Y. enterocolitica* subsp. *palearctica* 105.5R(r) with the equivalent loci of *D. dadantii* 3937 and *P. carotovorum* subsp. *carotovorum* PC1.

Bioinformatic analysis of the *Y. enterocolitica* subsp. *palearctica* 105.5R(r) (3/O:9 bio-serotype) genome (NCBI Reference Sequence: NC_015224.1) revealed the presence of an incomplete pectin degradation pathway compared to pectinolytic phytopathogens (Figure 1). The main difference is the lack of genes for secreted extracellular pectinases. However, the organization of gene clusters encoding some intracellular pectinolytic enzymes and transport systems involved in the uptake and catabolism of pectin derivatives is quite similar in the genomes of *Dickeya, Pectobacterium* species, *Y. enterocolitica* and in other pathogenic *Yersiniae* (Rodionov et al., 2004). The genome of *Y. enterocolitica* contains the paralogous genes *kdgM1* and *kdgM2* encoding the proteins KdgM1 and KdgM2, respectively, which are highly similar to one another (62% identity) and to the KdgM (65% for both proteins) and KdgN (57% for both proteins) oligogalacturonate-specific OM channels of *D. dadantii* (Figure S1 and Additional File 1). Interestingly, the *Y. enterocolitica kdgM1* gene is situated within a cluster of genes involved in the transport of OGAs into the cytoplasm, as is *kdgM*, its homolog in the *D. dadantii* genome (Rodionov et al., 2004). The *Y. enterocolitica kdgM2* gene is situated within a cluster of genes involved in OGA degradation, i.e., upstream from the gene pair *pelP-sghX*, respectively encoding pectate lyase PelP and the periplasmic polygalacturonate-binding protein SghX. The equivalent to *kdgM2* in *D. dadantii, kdgN*, is located in the vicinity of *ompW*, encoding a putative porin (Collao et al., 2013). The *kdgR* locus encoding repressor KdgR is similarly arranged in the genomes of *Y. enterocolitica* and the *Dickeya* and *Pectobacterium* species, being linked to downstream ORF *ogl*, which encodes an enzyme responsible for the cleavage of pectic dimers (Rodionov et al., 2004; Figure 1). The amino acid sequences of the KdgR regulators of *Y. enterocolitica* and *Dickeya* share 88% identity (Figure S2).

### Identification of the protein KdgM2 in the outer membrane of the *Y. enterocolitica ompR* deletion mutant

**Figure 2:**
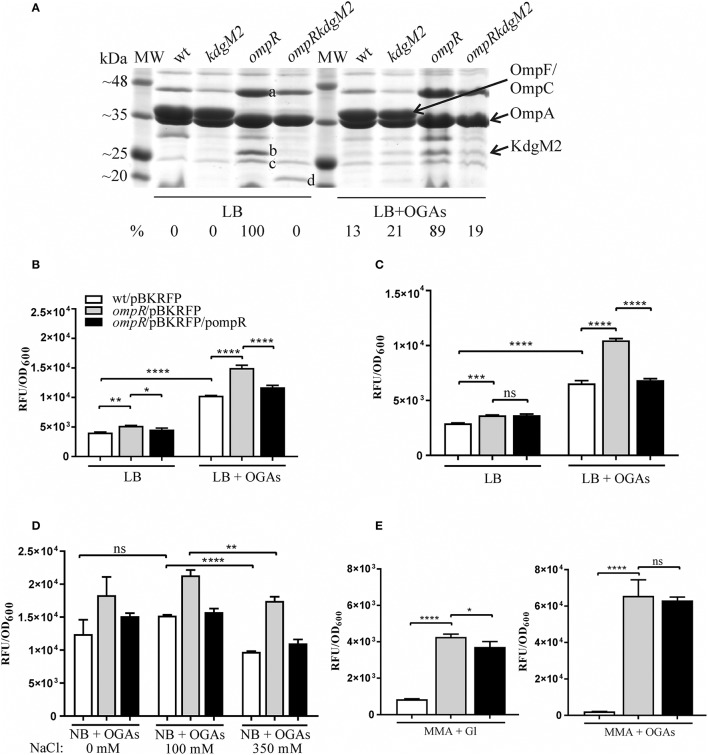
OmpR-dependent inhibition of KdgM2 production **(A)** and *kdgM2* expression **(B–E)**. **(A)** Production of KdgM2 was analyzed in outer membrane fractions prepared from bacterial cells grown overnight in LB medium without or with added OGAs at 26°C. The Coomassie blue-stained SDS-PAGE gel shows OMPs isolated from wild-type strain Ye9, *kdgM2* mutant MN1, *ompR* mutant AR4, and *ompRkdgM2* double mutant AR10. The relative intensities of the KdgM2 bands compared to the *ompR* mutant strain in LB (which was set to 100%) are indicated. The protein bands marked a, b, c, and d were excised and identified using LC-MS/MS. MW—molecular weight standards (3-Color Prestained Protein Marker, DNA-Gdańsk). The 12% SDS-polyacrylamide gel shown is representative of the results of an experiment performed several times. **(B–E)** RFP fluorescence intensity of strains Ye9N (wild-type), AR4 (*ompR* mutant) and complemented AR4 (*ompR*/pompR) containing a *kdgM2*′-′*rfp* translational fusion expressed from plasmid pBKRFP. Strains were cultivated in LB medium without or with OGAs at 26°C to exponential **(B)** or stationary **(C)** phase, and RFP fluorescence was measured. The effect of osmolarity **(D)** was analyzed by culturing strains at 26°C to exponential phase (2 h incubation) in NB medium with OGAs (0 mM NaCl) and supplemented with 100 mM or 350 mM NaCl (high osmolarity). **(E)** Strains were cultivated in MMA medium with glycerol (Gl) or with OGAs at 26°C to exponential phase. The data represent mean fluorescence activity values normalized to the OD_600_ of the culture (± standard deviation) from two independent experiments performed in triplicate. Significance was calculated using one-way ANOVA [ns (non-significant) *P* > 0.05, ^*^P < 0.05, ^**^P < 0.01, ^***^P < 0.001, ^****^P < 0.0001].

A comparative proteomic LC-MS/MS analysis of outer membranes prepared from wild-type *Y. enterocolitica* Ye9 (bioserotype 2/O:9) and the isogenic Δ*ompR*::Km mutant strain AR4 (Brzostek et al., 2003) revealed a number of differentially expressed proteins (Nieckarz et al., 2016). The most striking OmpR-dependent change was the strong upregulation of the protein KdgM2, a member of the KdgM family of oligogalacturonide-specific porins, initially described in *D. dadantii*. When grown in LB medium at 26°C, the *ompR* mutant strain exhibited a more than 100-fold increase in the level of this protein, indicating a major role for OmpR in the repression of KdgM2 production. To confirm this finding, SDS-PAGE analysis of OMPs isolated from *Y. enterocolitica* strains differing in their OmpR content, grown in LB medium without or with added OGAs (as an inducer) at 26°C, was performed (Figure 2A). Comparison of the OMP profiles revealed significant alterations in that of *ompR* mutant AR4 compared to the wild-type Ye9. Apart from the known lack of OmpC/OmpF porins (Brzostek and Raczkowska, 2007) at least three proteins showed increased abundance in the OM of the *ompR* mutant. One 48-kDa protein band and two bands at around 25 kDa were excised from the *ompR* mutant gel lane and analyzed by mass spectrometry. The 48-kDa band was identified as a homolog of *E. coli* maltoporin LamB, which is required for maltose and maltodextrin uptake (Boos and Schuman, 1998), (Figure 2A, band a). One of the bands at ~25 kDa was the protein KdgM2 (Figure 2A, band b), while the other, migrating to a position just below KdgM2, corresponded to the MltA-interacting protein MipA (Figure 2A, band c) (Vollmer et al., 1999). To confirm the OmpR-dependent negative regulation of KdgM2, *kdgM2* deletion mutants were constructed in both the wild-type strain Ye9N and the *ompR* mutant AR4, generating strains MN1 and AR10, respectively. SDS-PAGE analysis (Figure 2A) revealed that the KdgM2 protein band was absent from the gel profile of the *ompRkdgM2* double mutant (strain AR10) and this was accompanied by the appearance of a band at about 20 kDa. LC-MS/MS analysis of this band revealed a protein highly similar to the *E. coli* porin OmpW (Figure 2A, band d), that may be involved in the protection of bacteria against various forms of environmental stress (Hong et al., 2006).

The KdgM2 band was not visible in the gel profile of wild-type Ye9 grown in LB medium. However, following growth of this strain in LB with added OGAs, a band appeared at the position expected for KdgM2. Thus, the presence of OGAs derepressed *kdgM2* expression. The induction of oligogalacturonide-specific porins in the presence of pectin derivatives was reported previously in *D. dadantii* (Blot et al., 2002; Condemine and Ghazi, 2007). Interestingly, under derepressed conditions (LB+OGAs), a faint band appeared at the position of KdgM2 in the gel profiles of both the strain lacking KdgM2 (strain MN1) and the *ompRkdgM2* double mutant (strain AR10). This finding suggested that the expression of other OM oligogalacturonide-specific porins might be induced in *Y. enterocolitica* by OGAs, i.e., released from KdgR repression. In the view of our data presented below, we presume that the faint band corresponds to KdgM1, the second OGA-specific porin being under KdgR repression in *Y. enterocolitica*.

Taken together, the results of this SDS-PAGE analysis suggested that OmpR acts to reduce the level of KdgM2 directly and/or indirectly.

### OmpR negatively regulates the expression of *kdgM2*

The OmpR-dependent regulation of *kdgM2* was examined using a *kdgM2*′-′*rfp* translational fusion expressed from plasmid pBKRFP. Expression of this fusion was examined by quantifying RFP fluorescence in both the wild-type and *ompR* mutant strains carrying pBKRFP, following growth to exponential and stationary phase in LB without or with added OGAs as an inducer (Figures 2B,C). Compared to wild-type strain Ye9N, the *ompR* mutant displayed a ~1.3-fold increase in *kdgM2* expression when cultured in LB alone to either growth phase. When the LB was supplemented with OGAs, expression of the fusion in the wild-type strain was upregulated 2.5-fold and 2.3-fold in the exponential and stationary phases, respectively, indicating the release from the repressive activity of KdgR. In the *ompR* mutant grown in the presence of OGAs, the expression of *kdgM2* was ~1.5-fold higher than that observed in the wild-type strain. To confirm that the lack of OmpR leads to derepression of *kdgM2*, plasmid pHR4 carrying the wild-type *ompR* allele was used to complement the *ompR* mutation in strain AR4. This caused a clearly visible reduction in the expression of *kdgM2* in LB+OGAs, indicating that OmpR negatively regulates *kdgM2*. The lack (stationary phase) or only slightly visible (exponential phase) effect of complementation observed in LB medium alone (i.e., under KdgR-repressed conditions) suggested that OmpR might influence *kdgM2* expression in different ways.

The EnvZ/OmpR regulatory system has been shown to be involved in the osmoregulation of porin expression (Pratt et al., 1996). Thus, we were curious to see if *kdgM2* expression is subject to such regulation. The expression of the *kdgM2*′-′*rfp* fusion was therefore tested in strains grown in NB medium+OGAs without additions or supplemented with 100 mM or 350 mM NaCl (high osmolarity) (Figure 2D). A decreased level of RFP fluorescence was found in the wild-type strain after exposure to high osmolarity. This effect was also observed in the *ompR* mutant, suggesting that osmoregulation of *kdgM2* is independent of OmpR and may involve other regulatory mechanisms.

**Figure 3:**
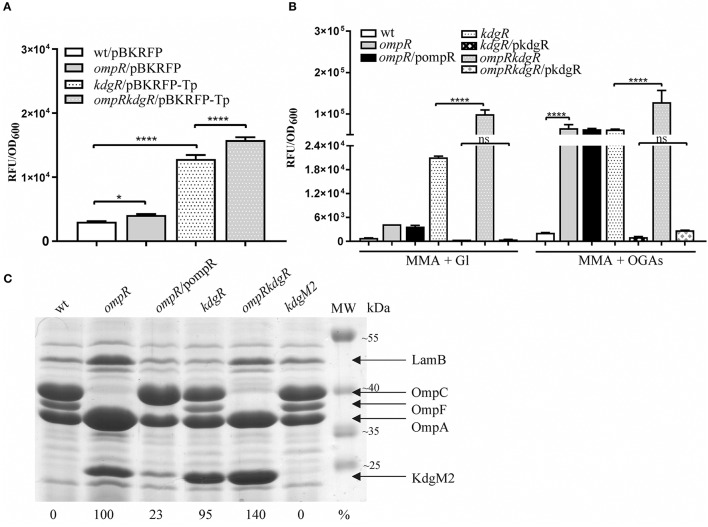
Complex regulation of *kdgM2* expression **(A,B)** and KdgM2 porin production **(C)** by OmpR and KdgR regulators. **(A,B)** RFP fluorescence intensity of strains Ye9N (wild-type), AR4 (*ompR* mutant), ES1 (*kdgR* mutant) and AR11 (*ompRkdgR* mutant) containing a *kdgM2*′-′*rfp* translational fusion expressed from plasmids pBKRFP or pBKRFP-Tp. Strains were cultivated in LB medium **(A)** or MMA+Gl and MMA+OGAs **(B)** at 26°C to exponential phase. Data represent mean fluorescence activity values normalized to the OD_600_ of the culture (±standard deviation) from two independent experiments performed in triplicate. Significance was calculated using one-way ANOVA [ns (non-significant) *P* > 0.05, ^*^P < 0.05, ^****^P < 0.0001]. **(C)** Absence of OmpR and KdgR independently leads to increased KdgM2 abundance. SDS-PAGE analysis was used to compare KdgM2 protein levels in the OM protein profiles of wild-type strain Ye9, the *ompR* mutant AR4, *ompR/*pompR (trans-complemented strain AR4), the *kdgR* mutant ES1, the *ompRkdgR* mutant AR11 and the *kdgM2* mutant MN1. The percentages indicate the KdgM2 band intensities in the tested strains relative to that in the *ompR* mutant AR4. The analyzed OM fractions were isolated from bacterial cells grown overnight in LB medium at 26°C. The positions of the LamB, OmpC, OmpF, OmpA, and KdgM2 proteins are indicated. MW – molecular weight standards (Thermo Scientific PageRuler Prestained Protein Ladder). The Coomassie blue stained 10% SDS-polyacrylamide gel shown is representative of the results of an experiment performed in triplicate.

Since the upregulation effect on *kdgM2* expression mediated by *ompR* deletion in the strains grown in LB medium was not as strong as anticipated (only ~1.3-fold), we examined expression of the *kdgM2*′-′*rfp* fusion in strains grown to exponential phase in minimal medium (MMA) supplemented with glycerol or OGAs as the carbon source. As seen in Figure 2E (and later in the text, Figure 3B), induction of *kdgM2* expression in the wild-type strain Ye9N grown in MMA+OGAs (~2.3-fold) was similar to that observed in LB+OGAs (~2.5-fold). Compared to the parent strain Ye9N, the *ompR* mutant expressed 4.5-fold more *kdgM2* in MMA+glycerol and 30-fold more in MMA+OGAs. No effect of complementation of the *ompR* mutation was observed in MMA+OGAs and only slightly visible effect was noted in MMA+ Gl (i.e., under KdgR-repressed conditions). Together these results demonstrated that when grown in either LB or MMA the *ompR*-negative strain exhibited a significantly elevated level of *kdgM2* expression and KdgM2 production compared to the parental strain. This phenotype was strongest in MMA supplemented with OGAs. From these data it may be speculated that OmpR is involved in the negative regulation of *kdgM2* expression. However, the complementation analysis indicated that the link between KdgM2 and OmpR might be more complex, and an optimal concentration of phosphorylated OmpR may be required to control the amount of KdgM2 protein. We cannot rule out the possibility that complementation by the *ompR* gene in multicopy leads to an excess of OmpR relative to EnvZ. Thus, the ratio of molecules of the kinase EnvZ to those of the substrate OmpR may be greatly imbalanced, which may influence the expression of *kdgM2*.

### Construction and characterization of *kdgR*-deficient *Y. enterocolitica* strains

Data showing upregulation of *kdgM2* caused by the *ompR* mutation and induction of *kdgM2* in the presence of OGAs led us to assume that apart from OmpR, the repressor KdgR inhibits *kdgM2* expression. To dissect these effects and, in addition, to eliminate the problem of the transport of inducing OGAs, we examined OmpR-mediated regulation of *kdgM2* expression in the absence of KdgR. The *kdgR* gene was deleted in both the wild-type strain Ye9N (*kdgR* mutant ES1) and in the *ompR* mutant AR4 (*ompRkdgR* mutant AR11). To study *kdgM2* expression in these strains, the plasmid pBKRFP-Tp carrying the *kdgM2*′-′*rfp* translational fusion was introduced. The presence of the gentamicin (Gm) resistance cassette in the mutant *kdgR* made it necessary to insert a trimethoprim (Tp) cassette into pBKRFP carrying the *kdgM2*′-′*rfp* fusion. RFP fluorescence was examined in cultures grown at 26°C in LB (Figure 3A) and MMA medium (Figure 3B). Data for the strains grown to exponential phase in LB medium (Figure 3A) revealed that in strain ES1 the absence of only KdgR in the wild-type background resulted in a 4-fold increase in the expression of *kdgM2* (for comparison a 2.5-fold increase was observed in the wild-type in the presence of OGAs). The absence of only OmpR in strain AR4 resulted in a 1.4-fold increase in *kdgM2* expression. In strain AR11, the lack of KdgR in the *ompR* mutant background resulted in significantly higher *kdgM2* expression than that detected in the strains with single *ompR* (3.9-fold) or *kdgR* (1.2-fold) mutations. The same correlation was obtained with the strains grown in LB to stationary phase (data not shown).

The effect of the *ompR* mutation on *kdgM2* expression was also examined in strains grown in minimal medium to exponential phase. In MMA+glycerol, the upregulation of *kdgM2* in the *ompR*-deficient strain was 4.5-fold, while in the *kdgR*-deficient strain it was 22-fold (Figure 3B). In the strain containing both regulatory mutations *ompRkdgR*, the level of *kdgM2* expression was significantly higher (100-fold) than in either single mutant, suggesting a synergistic mode of regulation. These data led us to the hypothesis that this growth condition triggers a cascade of derepression and activation of the *kdgM2* gene, allowing its maximal expression. The addition of OGAs to the minimal medium produced further upregulation of *kdgM2* expression in the *ompR* (30-fold) and *kdgR* (29-fold) strains. The additive effect of *kdgR* and *ompR* mutations was observed in *ompRkdgR* mutant (60-fold) suggested that KdgR and OmpR regulate *kdgM2* expression independently. Complementation of the *kdgR* mutation with the wild-type *kdgR* allele expressed from plasmid pHSG575 caused a very strong reduction in *kdgM2* expression back to the wild-type level. Interestingly, the expression of *kdgR in trans* in the *ompRkdgR* mutant almost completely abolished the positive regulatory effect of the *ompR* mutation.

Taken together, these results confirmed the role of KdgR as a repressor of *kdgM2* expression and showed that OmpR likely contribute to the negative regulation of *kdgM2* expression irrespective of KdgR. The observed induction of *kdgM2* by OGAs in minimal medium in the absence of KdgR suggested that other regulatory factors acting independently of KdgR (also active in the *kdgR* mutant) might be responsible for the induction by intermediates of OGA catabolism. The inducing power of OGAs in the absence of *kdgR* was reported previously in a study analyzing the production of pectate lyase in *kdgR* mutants in the presence of other mutations (Hugouvieux-Cotte-Pattat and Robert-Baudouy, 1989). Finally, the medium-dependent expression of *kdgM2* could be the consequence of variations in the respective level/activity of OmpR and KdgR. Previously, differences in *kdgN* porin expression were observed during the growth of *D. dadantii* in rich LB medium and M63 minimal medium, but the underlying regulatory mechanism was not identified (Condemine and Ghazi, 2007).

### The level of KdgM2 porin in the outer membrane of *Y. enterocolitica* is influenced by regulators OmpR and KdgR

We determined the effect of KdgR and OmpR on the KdgM2 protein content in the OM by examining wild-type Ye9 and isogenic strains with *kdgR, ompR*, and *ompRkdgR* mutations (Figure 3C). The KdgM2 protein band was absent from the gel profile of the wild-type Ye9. strain grown in LB medium and appeared in an OM fraction from cells of *kdgR* mutant ES1 confirmed that KdgR represses expression of this protein. The effect of a lack of OmpR was examined in the presence (*ompR* mutant, strain AR4) and absence (*ompRkdgR* mutant, strain AR11) of a functional *kdgR* gene. The *ompR* mutant AR4 exhibited an increased level of KdgM2 expression compared to the parental wild-type strain Ye9, confirming the observation that initiated our studies and the data from genetic analyses. The level of the KdgM2 in the *ompR* mutant was similar to that observed in the *kdgR* mutant strain. In the double *ompRkdgR* mutant strain AR11, the level of KdgM2 was significantly increased compared to the single *ompR* and *kdgR* mutants (for both ~1.4-fold). This result suggested that the lack of OmpR increased the biosynthesis of KdgM2 independently of KdgR. When the wild-type allele of *ompR* was introduced into mutant AR4 *in trans* on plasmid pBR3, the production of KdgM2 decreased significantly, although not to the wild-type level. These data demonstrated the negative effect of OmpR in controlling the level of KdgM2 and corroborated the results of the *kdgM2* reporter fusion experiments described above.

### Increased pel activity in the *ompR* and *kdgR* mutants

**Figure 4:**
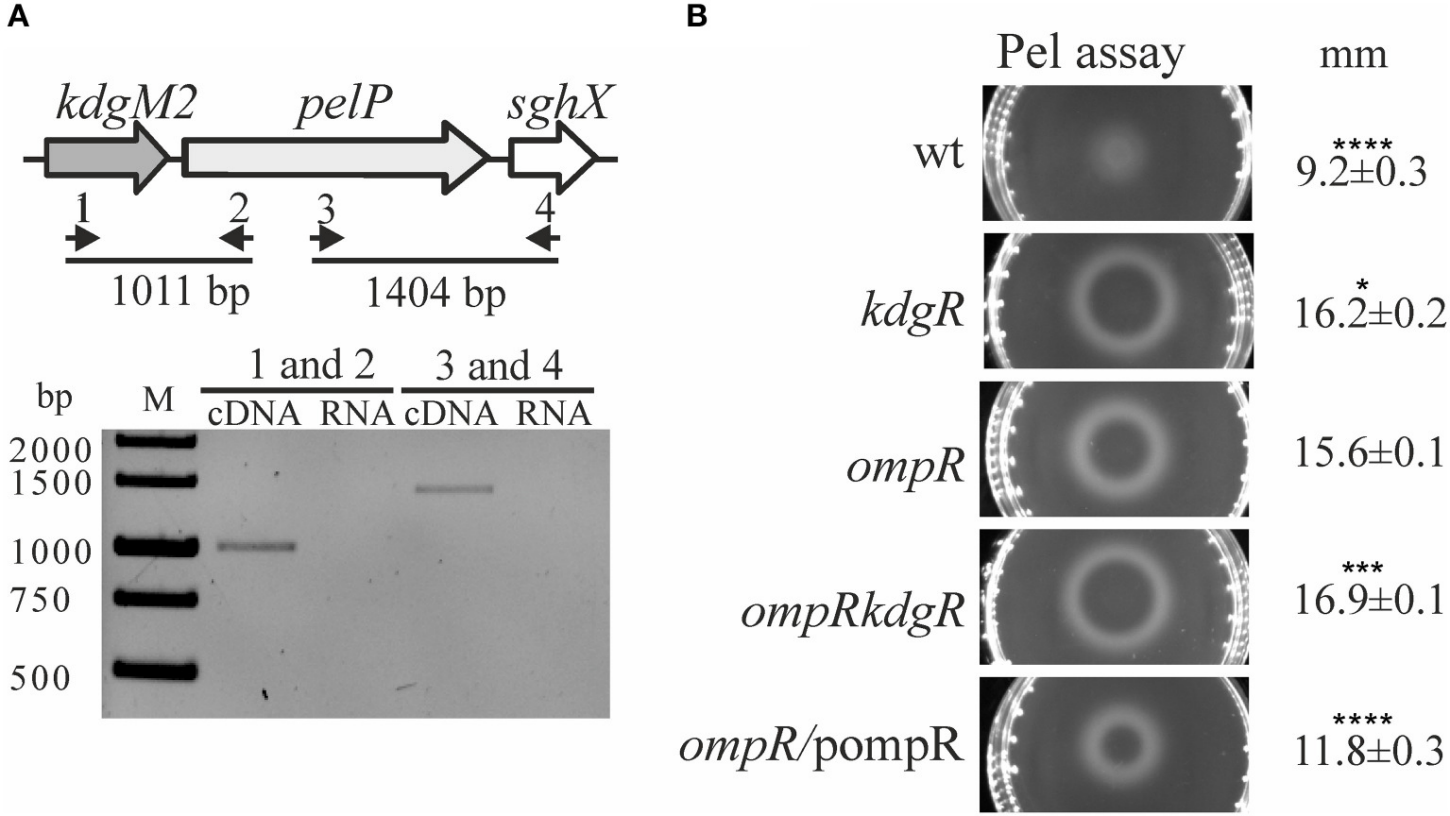
Effect of OmpR and KdgR on pectate lyase (PelP) production. **(A)** To determine whether the *kdgM2, pelP*, and *sghX* genes are organized in an operon, two pairs of primers were used in RT-PCR analysis (1–RTkdgMpelP1/2–RTkdgMpelP2 and 3–RTpelPsghX1/4–RTpelPsghX2) (**A**, upper panel). RT-PCR analysis of the *kdgM2-pelP-sghX* operon (**A**, lower panel). Total RNA isolated from strain Ye9 grown in LB medium at 26°C was DNase treated and then reverse transcribed into cDNA with pairs of primers shown in **(A)** upper panel. M – molecular size marker GeneRuler 1 kb DNA Ladder; bp. RNA was used as the template in negative control reactions. **(B)** Changes in pectate lyase (PelP) production in the tested *Y. enterocolitica* mutants lacking OmpR, KdgR or both regulatory proteins. Periplasmic PelP activity was determined in a semi-quantitative manner by measuring the diameter of haloes on plates containing OGAs and Ca^2+^, 48 h after inoculation with osmotic shock fluid obtained from exponential phase *Y. enterocolitica* cultures grown in LB medium at 26°C. PelP production was compared in the following strains: Ye9 (wild-type), ES1 (*kdgR* mutant), AR4 (*ompR* mutant), AR11 (*ompRkdgR* mutant), and complemented AR4 (*ompR*/pompR). The data represent mean values with standard deviations, obtained in at least three independent experiments. Significance was calculated using one-way ANOVA (^*^P < 0.05, ^***^P < 0.001, ^****^P < 0.0001). *P*-values were calculated using (assuming unequal variances) comparing test strains to the AR4 strain.

It was previously shown that *Y. enterocolitica* produces a few intracellular pectate lyases, i.e., enzymes involved in OGA degradation, including periplasmic PelP (*Ye*PL2A), cytoplasmic PelW (*Ye*PL2B) and Ogl (*Ye*OGL) (Abbott and Boraston, 2007, 2008). It has been postulated that *kdgM2, pelP* and *sghX*, encoding porin KdgM2, pectate lyase PelP and putative periplasmic polygalacturonate binding protein SghX, respectively, might be organized in an operon in *Yersiniae* (Rodionov et al., 2004). To confirm this arrangement in *Y. enterocolitica* wild-type strain Ye9, RT-PCR analysis was performed with cDNA using primer pairs specific for the *kdgM2-pelP-sghX* mRNA (Figure 4A, upper panel). The results of this analysis showed that these three genes are co-transcribed as a polycistronic mRNA (Figure 4A, lower panel). Thus, in addition to porin KdgM2, the production of proteins PelP and SghX might also be regulated by OmpR.

To examine whether the lack of OmpR or KdgR influenced the production of periplasmic pectate lyase PelP by *Y. enterocolitica*, we used the pectate lyase (Pel) plates assay (Figure 4B). The presence of PelP lyase in periplasmic fluid (released by osmotic shock) of the wild-type (Ye9), *ompR* mutant (AR4), *kdgR* mutant (ES1) and the *ompRkdgR* mutant (AR11) was tested. In the wild-type Ye9, pectate lyase production was low. In contrast, high pectate lyase activity was observed in the *kdgR* mutant, confirming the repressive activity of KdgR. The *ompR* mutant (AR4) exhibited an increased level of pectate lyase production compared to wild-type cells, which was comparable to that of the *kdgR* mutant strain (ES1). Pectate lyase activity in the double *ompRkdgR* mutant was slightly higher than in the single mutants. Complementation of the *ompR* mutation using pBR3 caused a reduction in PelP activity (Figure 4B). The changes in PelP production in the tested *Y. enterocolitica* mutants lacking OmpR, KdgR or both regulatory proteins, reflected the level of porin KdgM2 present in these mutants. These results corroborated our finding that *kdgM2* and *pelP* are organized in an operon whose promoter is negatively regulated by OmpR and KdgR.

### OmpR positively regulates the expression of *kdgM1*, a paralog of *kdgM2*

**Figure 5:**
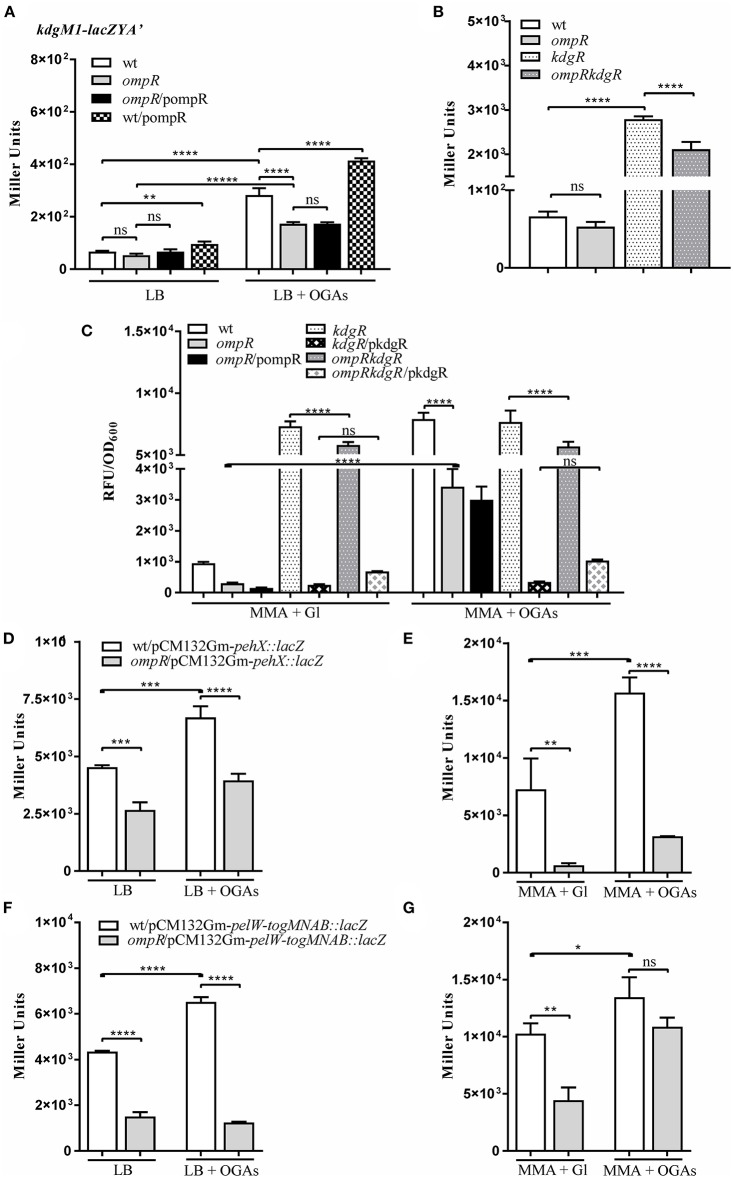
OmpR promotes expression of the genes/operon of the KdgR regulon: *kdgM1* **(A–C)** *pehX* **(D,E)** and *pelW-togMNAB* **(F,G)**. **(A–C)** β-galactosidase activity of strains carrying a chromosomal *kdgM1-lacZYA*′ transcriptional fusion, with or without OmpR or/and KdgR: Ye9NK1 (wild-type, wt), AR4K1 (*ompR* mutant), AR4K1/pompR (complemented *ompR* mutant, *ompR*/pompR) and Ye9NK1/pompR (wild-type overproducing OmpR, wt/pompR), ES1K1 (*kdgR* mutant) and AR11K1 (*ompRkdgR* mutant). β-galactosidase activity of Ye9N (wild-type) and AR4 (*ompR* mutant) strains containing *pehX* **(D,E)** and *pelW-togMNAB* **(F,G)** transcriptional fusions with *lacZ* expressed from plasmids pCM132Gm-*pehX::lacZ* and pCM132Gm-*pelW-togMNAB::lacZ*, respectively. Strains were grown at 26°C without or with OGAs, to stationary phase in LB medium **(A,B,D,F)** or to exponential phase in MMA medium **(C,E,G)**. The data represent mean β-galactosidase activity values (Miller units) with the standard deviation from at least two independent experiments, each performed using at least triplicate cultures of each strain. Significance was calculated using one-way ANOVA (ns [non-significant] *P* > 0.05, ^*^P < 0.05, ^**^P < 0.01, ^***^P < 0.001, ^****^P < 0.0001).

Bioinformatic analysis revealed the presence of another gene encoding an oligogalacturonide-specific porin of the KdgM family in the genome of *Y. enterocolitica*, i.e., *kdgM1* (Additional File 1). To verify whether OmpR or KdgR control *kdgM1* transcription, we examined the expression of a *kdgM1-lacZYA*′ chromosomal transcriptional fusion in the wild-type strain (Ye9NK1), *ompR* mutant (AR4K1), *kdgR* mutant (ES1K1) and *ompRkdgR* mutant (AR11K1) (Figures 5A–C). First, we measured the β-galactosidase activity in LB medium, in strains differing in their OmpR content, in the absence and presence of OGAs (Figures 5A,B). The expression of the transcriptional fusion in cells of the wild-type strain grown in LB medium to stationary phase was very low and it was increased 4.3-fold by the addition of OGAs. In non-inducing conditions, we did not observe any difference between the wild-type strain and the *ompR* mutant. In LB supplemented with OGAs, the activity of the *kdgM1* promoter in the *ompR* mutant was increased, but to a lesser extent than in the wild-type strain. To confirm the positive OmpR-dependent regulation of *kdgM1*, plasmid pBR3 carrying the wild-type *ompR* allele was used to complement the *ompR* mutation in strain AR4K1. However, complementation did not produce any change in the level of reporter gene expression. We next introduced pBR3 into the wild-type strain (Ye9NK1) and found that overexpression of OmpR caused a 1.5-fold increase in β-galactosidase activity compared to the wild-type strain alone (Figure 5A). Notably, the lack of KdgR caused a 42-fold upregulation of the *kdgM1* promoter in strain ES1K1, suggesting that KdgR strongly represses KdgM1 production in LB medium (Figure 5B). We could not rule out the possibility that in the absence of the KdgR repressor, putative activators may increase *kdgM1* expression under these growth conditions. In the *ompRkdgR* mutant AR11K1 we observed a significant decrease in reporter gene expression (1.3-fold) compared to the *kdgR mutant* (ES1K1). The positive influence of OmpR on *kdgM1* expression was also seen in strains grown to exponential phase in LB medium (data not shown).

To shed light on the OmpR-dependent regulation of *kdgM1* we investigated the expression of the *kdgM1-lacZYA*′ fusion in *Y. enterocolitica* strains grown to exponential phase in minimal medium. The 11-fold upregulation of *kdgM1* in the wild-type strain cultured in MMA+OGAs contrasted with the weak expression in MMA+glycerol (Figure 5C). Almost the same increase (10-fold) was observed in the *kdgR* mutant independently of the growth conditions (MMA medium supplemented with glycerol or OGAs), confirming the role of KdgR as a repressor of *kdgM1*. In addition, introduction of the wild type *kdgR* allele expressed from plasmid pBBR1MCS-3 reduced *kdgM1* expression in MMA+glycerol. However, the same effect was observed in the presence of the inducer (OGAs). Possibly the intracellular concentration of the direct inducer 2-keto-3-deoxygluconate, a product of OGA degradation, was insufficient to saturate high levels of KdgR expressed from the multicopy plasmid. Inactivation of the *ompR* gene decreased the expression of the *kdgM1* fusion in MMA+glycerol and MMA+OGAs (Figure 5C) 2.6-fold and 2.4-fold, respectively, in both the wild-type and the *kdgR* mutant background. These results revealed the role of OmpR in the positive regulation of *kdgM1* expression, independently of the presence of KdgR regulator. Unfortunately, we were unable to detect any complementary effect of the *ompR* mutation. Variations in osmolarity caused no change in the level of *kdgM1* expression examined in exponential phase cultures (data not shown).

### OmpR influences the expression of other members of the KdgR regulon

To examine whether OmpR might affect the expression of genes of the KdgR regulon in *Y. enterocolitica*, we constructed plasmid-borne *lacZ* transcriptional fusions with the promoter regions of the *pehX* gene encoding the periplasmic polygalacturonase PehX and the *pelW-togMNAB* operon encoding the cytoplasmic exopolygalacturonate lyase PelW and oligogalacturonide transport system TogMNAB (Abbott and Boraston, 2008). The resulting constructs pCM132Gm-*pehX::lacZ* and pCM132Gm-*pelW-togMNAB::lacZ* were introduced into the wild-type strain Ye9N and the *ompR* mutant AR4. Based on β-galactosidase activity measurements, we found that *pehX* and *pelW-togMNAB* expression were both 1.5-fold higher in strain Ye9N grown at 26°C to stationary phase in LB medium supplemented with OGAs, compared to LB alone (Figures 5D,F). This confirmed the OGA-inducible nature of both promoters and suggested the participation of KdgR in their repression. The expression of both *pehX* and *pelW-togMNAB* was higher in the wild-type strain compared to the *ompR* mutant AR4 when they were grown in LB medium (1.7-fold and 2.9-fold, respectively) (Figures 5D,F), thus revealing the positive impact of OmpR on these genes. In the presence of inducer (OGAs), we still observed reduced *pehX* and *pelW-togMNAB* expression in the *ompR* mutant background. Expression of the *pehX* fusion in strains grown in LB to exponential phase was not significantly modified in the *ompR* mutant, while expression of the *pelW-togMNAB* fusion was equivalent in cells in both the exponential and stationary phases of growth (data not shown). Furthermore, the regulatory role of OGAs and OmpR in the upregulation of the *pehX* (Figure 5E) and the *pelW-togMNAB* (Figure 5G) was also observed in strains grown to exponential phase in MMA medium without or with OGAs. Interestingly, the OmpR-dependent regulation of the *pelW-togMNAB* expression was not observed in the presence of OGAs. In summary, OmpR seems to be a positive regulator of both studied transcriptional units. However, since no OmpR binding sites have been detected in either the *pehX* or the *pelW-togMNAB* regulatory regions, we presume that the positive influence of OmpR might result from some indirect effect that it exerts on their transcription.

### OmpR negatively regulates *kdgR* transcription

**Figure 6:**
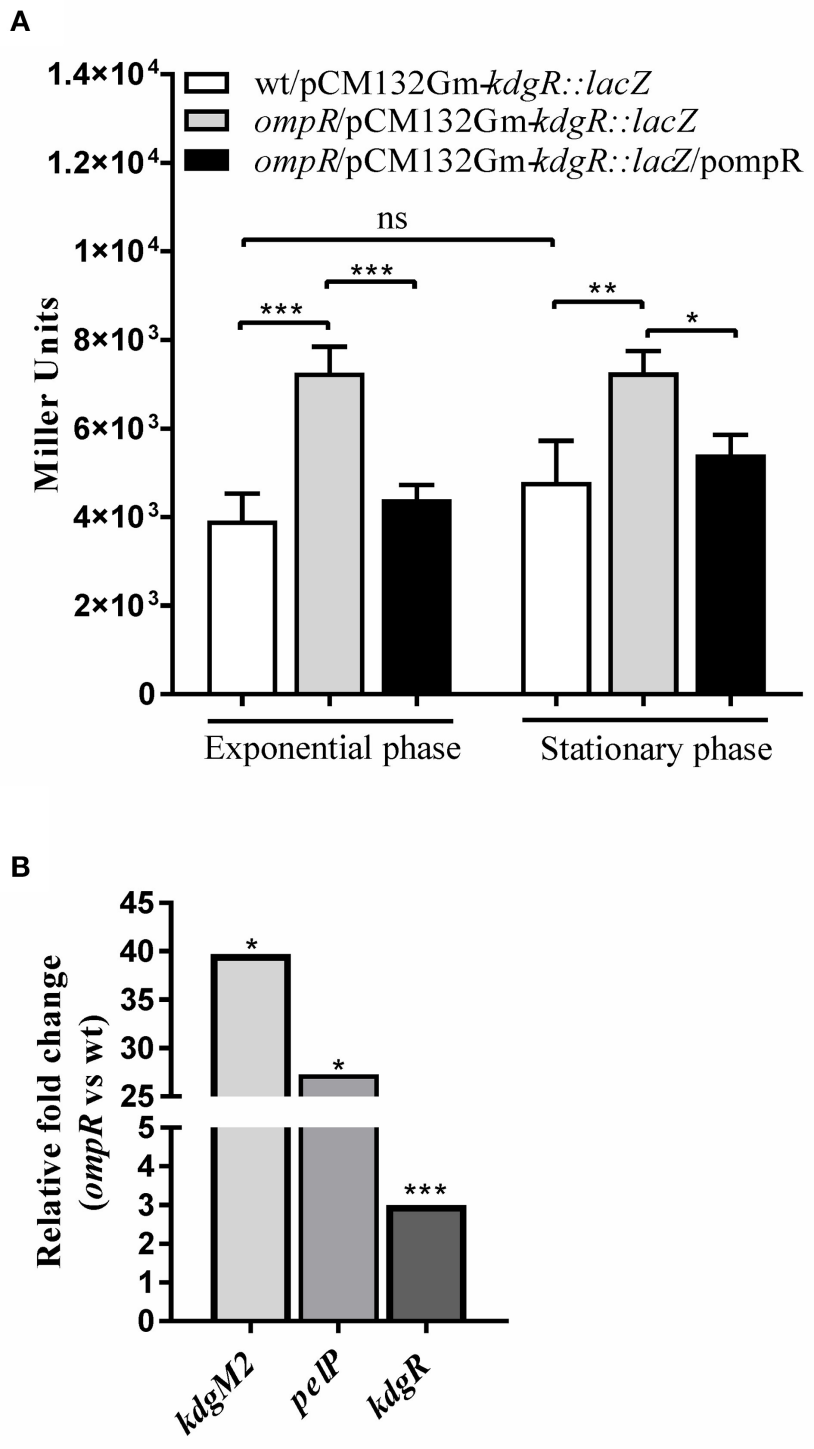
Effect of OmpR on *kdgR* expression using *lacZ* reporter fusions **(A)** and RT-qPCR analysis **(B)**. **(A)** β-galactosidase activity of strains Ye9N (wild-type), AR4 (*ompR* mutant) and complemented AR4 (*ompR*/pompR) containing a *kdgR*::*lacZ* transcriptional fusion expressed from plasmid pCM132Gm-*kdgR::lacZ*. Strains were grown to exponential and stationary phase in LB at 26°C and β-galactosidase activity was assayed. The data represent mean activity values (Miller units) with the standard deviation from two independent experiments, each performed using at least triplicate cultures of each strain. Significance was calculated using one-way ANOVA (ns [non-significant] *P* > 0.05, ^*^P < 0.05, ^**^P < 0.01, ^***^P < 0.001). **(B)** mRNA levels of *kdgR, kdgM2* and *pelP* were determined by quantitative real-time polymerase chain reaction (RT-qPCR analysis) from wild-type and *ompR* mutant strains grown in LB+OGAs to stationary phase at 26°C as described in Materials and Methods. Representative results from two independent experiments, performed in quadruplicate, are shown. The statistical significance of differences in transcripts of tested genes were analyzed using Student's *t*-test. Stars indicate statistically significant differences (^*^P < 0.05, ^***^P < 0.001).

To investigate the possible link between the OmpR and KdgR regulatory proteins, OmpR-dependent activity of the *kdgR* promoter was studied using a transcriptional fusion with a promoterless *lacZ* gene, constructed in plasmid pCM132Gm. The resulting construct pCM132Gm-*kdgR::lacZ* was introduced into the wild-type strain Ye9N and the *ompR* mutant AR4. The levels of expression of *kdgR*::*lacZ*, based on measurements of β-galactosidase activity, were determined for both strains grown to exponential and stationary phase in LB medium at 26°C (Figure 6A). The activity of the *kdgR* promoter in the *ompR* mutant was 1.9-fold (in exponential phase) and 1.5-fold (in stationary phase) higher than in the Ye9N strain. For complementation analysis, vector pBR3 carrying the entire *ompR* coding sequence was introduced into strain AR4/pCM132Gm-*kdgR::lacZ*. The introduction of *ompR in trans* restored *kdgR* expression to the wild-type level. These results suggested that OmpR negatively regulates *kdgR* expression. Data from the *kdgR::lacZ* reporter fusion were validated by evaluating the expression of *kdgR* in the wild-type Ye9 and the *ompR* mutant AR4 grown to stationary phase in LB+OGAs at 26°C, using quantitative real-time PCR (Figure 6B). In addition, as a control *kdgM2* and *pelP* transcript abundance was also assessed. Transcription of *kdgR* was up-regulated in the *ompR* mutant (3-fold), confirming that OmpR functions as a negative regulator of *kdgR*. In contrast, *kdgM2* and *pelP* were highly up-regulated in the *ompR* mutant, 39-fold and 27-fold, respectively. These results are consistent with those obtained for the *ompR* mutant with the *kdgM2::lacZ* reporter fusion and PelP activity test.

Taken together, these results demonstrated that besides its role in regulating *kdgM2, kdgM1, pehX* and *pelW-togMNAB* (shown above), OmpR modulates the expression of their regulator KdgR. Bearing in the mind that OmpR negatively regulates *kdgR* expression, the KdgM2 level may reflect the dual function of OmpR: having both a direct negative effect on *kdgM2* transcription and an indirect effect by repressing *kdgR*. The positive regulatory effect of OmpR on *kdgM1* may result from both its direct positive effect on *kdgM1* expression and the indirect effect of its inhibition of *kdgR* expression.

### Putative KdgR and OmpR binding sites in the promoter regions of *kdgM1, kdgM2-pelP-sghX* and *kdgR* of *Y. enterocolitica*

**Figure 7:**
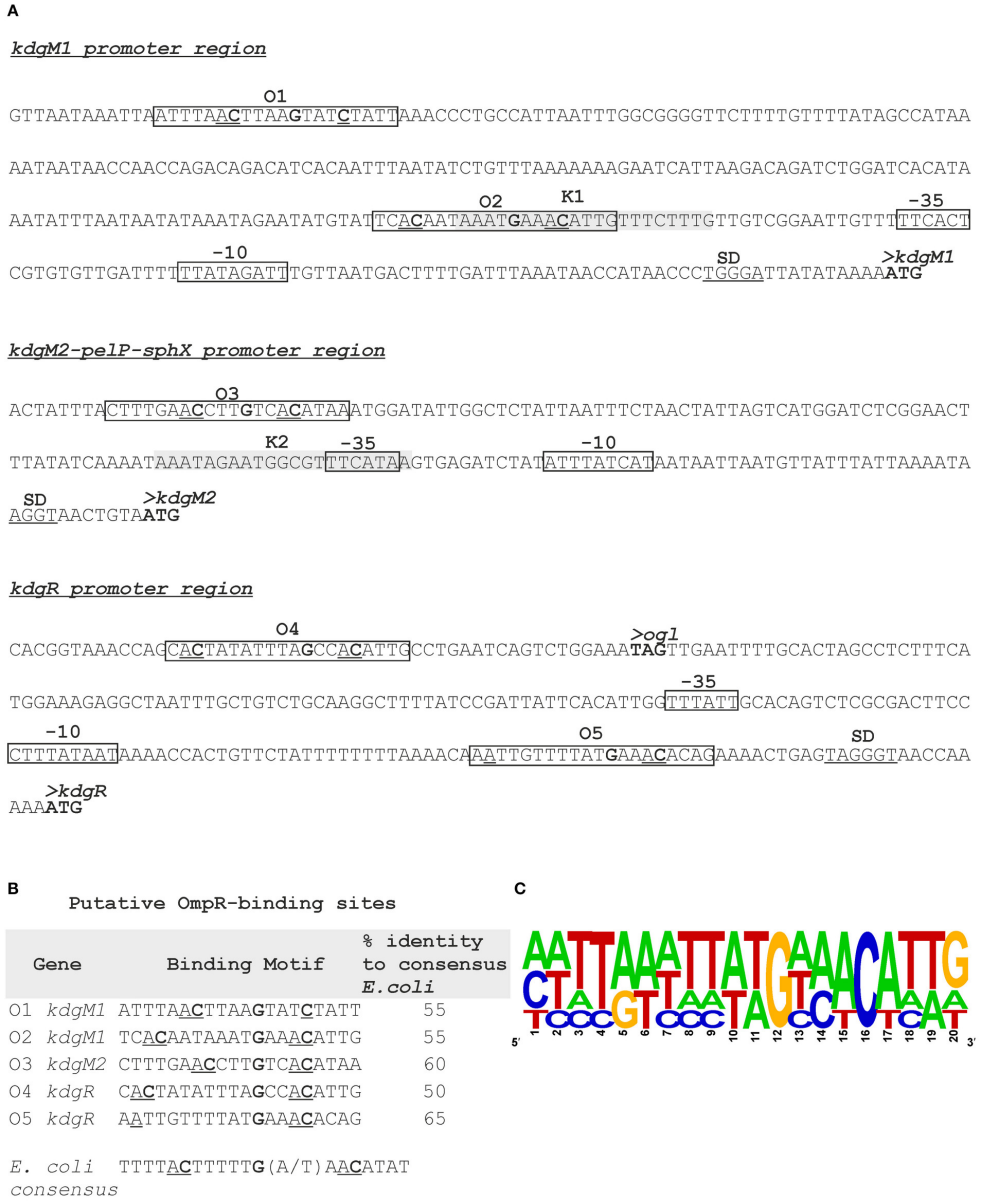
Putative OmpR-binding sites in the promoter regions of the *kdgM1, kdgM2-pelP-sghX* and *kdgR* genes of *Y. enterocolitica*. **(A)** The boxes indicate putative −35 and −10 promoter elements. The SD sequence is underlined. The start codon (ATG) of each gene is shown in bold. Putative OmpR binding sites identified by *in silico* analysis (O1-O5) are boxed. Potential binding sites for KdgR (K1, K2) are shaded gray. **(B)** Sequences of putative OmpR-binding sites in the promoters of the indicated *Y. enterocolitica* genes determined based on similarity to the *E. coli* consensus sequence (% identity values are shown). The central motif GXXAC or GXXXC and the AC or C nucleotides usually located about 10 nt away from the AC elements of the central motif are marked. **(C)** Logo motif (alignment) of putative OmpR-binding sites identified in the *Y. enterocolitica kdgM1, kdgM2-pelP-sghX* and *kdgR* regulatory regions. WebLogo (http://weblogo.berkeley.edu/logo.cgi) was used to obtain consensus sequence logos in which the height of individual letters represents the relative frequency of that particular nucleotide at a given position, and the number of letters in each stack indicates the degree of conservation at that position.

Putative KdgR and OmpR binding sites in the regulatory regions of selected KdgR regulon members were identified by *in silico* analysis (Figure 7). The promoters of the *kdgM1, kdgM2-pelP-sghX* and *kdgR* genes/operon were initially characterized using BPROM software. Then a search conducted using the *D. dadantii* consensus KdgR binding site sequence (AAATGAAACAnTGTTTCATTT, Rodionov et al., 2000, 2004) led to the identification of potential KdgR-binding site K1 close to the *kdgM1* promoter (90% identity to consensus) and K2 overlapping the *kdgM2-pelP-sghX* promoter (65% identity to consensus). No putative KdgR-binding sites were identified in the *kdgR* regulatory region. The consensus OmpR-binding sequence of *E. coli* (TTTTACTTTTTG(A/T)AACATAT, Maeda et al., 1991) was used to search for putative OmpR binding sites. Two predicted OmpR-binding sites were found in the *kdgM1* promoter region: O1 and O2, both with 55% identity to the consensus (Figures 7A,B). The putative O1 and O2 sites are respectively located between nucleotides −277 and −297, and −101 and −121 bp, upstream of the *kdgM1* ATG, with the second site overlapping the proposed KdgR-binding site. The location of this OmpR-binding site might be related to the role of OmpR as an antirepressor in the positive regulation of *kdgM1* transcription. One putative OmpR-binding site was detected in the *kdgM2-pelP-sghX* regulatory sequence: O3 with 60% identity to the consensus (Figures 7A,B), located between nucleotides −141 and −161 bp upstream of the *kdgM2* ATG. The results of this analysis indicated that *kdgM1* and *kdgM2-pelP-sghX* might be subject to dual regulation by KdgR and OmpR in *Y. enterocolitica*. Two potential OmpR-binding sites were also recognized in the *kdgR* regulatory region: sites O4 and O5 located between nucleotides −207 and −227 bp and −24 and −44 bp upstream of the *kdgR* ATG, with 50% and 65% identity to the consensus, respectively (Figures 7A,B). The results of Logo motif analysis of these putative *Y. enterocolitica* OmpR-binding sites are shown in Figure 7C. The presence of OmpR-binding sites in the *kdgR* regulatory region suggests a direct role for OmpR in regulating KdgR expression.

### OmpR directly regulates *kdgM1, kdgM2*, and *kdgR* expression in *Y. enterocolitica*

**Figure 8:**
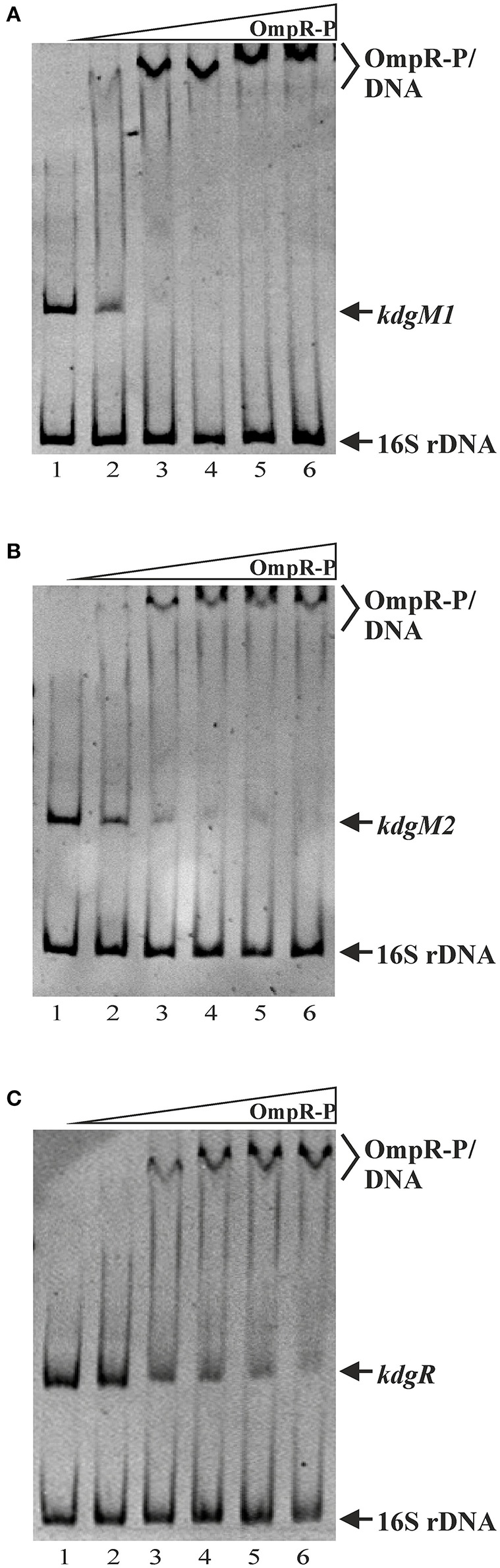
Binding of phosphorylated OmpR to the *kdgM1, kdgM2-pelP-sghX*, and *kdgR* regulatory regions examined using EMSAs. A range of concentrations of OmpR-P were incubated with DNA fragments representing the *kdgM1* (**A**, 544 bp), *kdgM2-pelP-sghX* (**B**, 500 bp) and *kdgR* (**C**, 441 bp) promoters which contain putative OmpR-binding sites. A fragment of 16S rDNA (304 bp) was included in each reaction mixture as a non-specific binding control. The DNA fragments were mixed with increasing concentrations of OmpR-P in lanes 1–6: 0, 0.167, 0.333, 0.500, 0.583, 0.667 μM. The identities of the bands resolved by electrophoresis on native 5% polyacrylamide gels are indicated.

As shown above, *in silico* analysis led to the identification of putative OmpR-binding sites in the promoter regions of the *Y. enterocolitica kdgM1* gene (2 sites), *kdgM2-pelP-sghX* operon (1 site) and *kdgR* gene (2 sites) (Figure 8). To verify whether OmpR directly binds to these sequence elements, electrophoretic mobility shift assays (EMSAs) were performed. A recombinant OmpR-His_6_ protein was expressed in *E. coli*, purified to homogeneity and phosporylated *in vitro* using acetyl-phospate. It was shown previously that OmpR can be phosphorylated by acetyl phosphate *in vivo* and *in vitro* (Shin and Park, 1995). DNA fragments representing the promoter region of the analyzed genes/operon, containing the putative OmpR-binding sites, and a fragment of 16S rDNA, as a non-specific binding control, were incubated with different amounts of the OmpR-P and these binding reactions were analyzed by electrophoresis in non-denaturing 5% polyacrylamide gels. As shown in Figure 8, OmpR-P was able to bind the upstream regions of *kdgM1, kdgM2-pelP-sghX*, and *kdgR*, but not the 16S rDNA fragment. Shifted complexes were clearly produced by interaction between the *kdgM1* and *kdgM2-pelP-sghX* fragments and phosphorylated OmpR present at a concentration of 0.167 μM (Figures 8A,B). Interestingly, the OmpR-P protein interacted with the *kdgR* promoter fragment with slightly lower affinity. A slower migrating nucleoprotein band appeared when a higher concentration of OmpR-P (0.333 μM) was incubated with the *kdgR* fragment (Figure 8C). Since our *in silico* analysis identified two putative OmpR-binding sites in the regulatory regions of *kdgM1* and *kdgR*, a stepwise shift in the nucleoprotein complexes might be expected in EMSAs. Indeed, we observed a slight stepwise shift when both promoter region fragments were used in an EMSA. To reveal the exact number of OmpR binding sites and the specific nucleotide sequence to which OmpR-P binds, a DNase I footprinting experiment would be necessary. Taken together, these results demonstrated that OmpR can specifically bind to the *kdgM1, kdgM2*-*pelP*-*sghX*, and *kdgR* promoter regions.

### Intracellular ROS production in response to short OGAs

**Figure 9:**
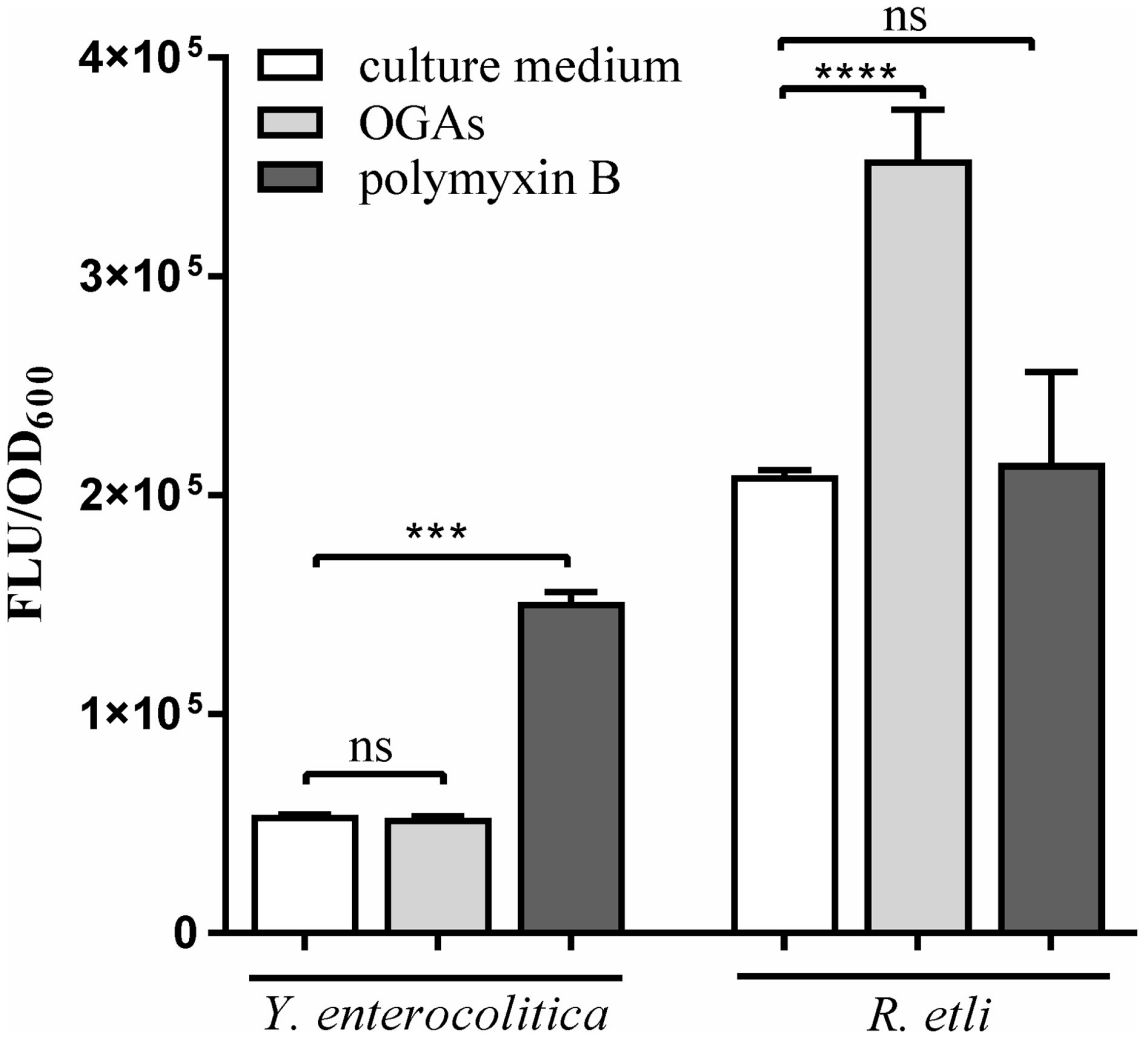
Effect of short oligogalacturonides (sOGAs) and polymyxin B on the production of ROS in *Y. enterocolitica* Ye9 and *R. etli* CE3. ROS formation was detected using the fluorescent dye H2DCF-DA. Strains were cultivated in LB medium (*Y. enterocolitica*) or in TY medium (*R. etli*) at 26°C to exponential phase and treated with sOGAs (50 μg/ml) or polymyxin B (25 μg/ml). Data represent mean fluorescence activity values normalized to the OD_600_ of the culture (± standard deviation) from experiments performed in triplicate. Significance was calculated using one-way ANOVA [ns (non-significant) *P* > 0.05, ^***^P < 0.001, ^****^P < 0.0001].

Short oligogalacturonides (sOGAs) penetrating the cells of phytopathogens are able to induce the generation and accumulation of reactive oxygen species (ROS) (Côté and Hahn, 1994; Ridley et al., 2001). sOGAs have been shown to evoke a transient accumulation of ROS in *Rhizobium leguminosarum* bv. *viciae* 3841 (Moscatiello et al., 2012). To investigate the effect of sOGAs on cells of *Y. enterocolitica* Ye9 and *Rhizobium etli* CE3 we analyzed intracellular ROS production following treatment with sOGAs by measurement of 2′,7′-dichlorodihydrofluorescein diacetate (H2DCF-DA) fluorescence (Figure 9). Short linear α-1,4-linked oligogalacturonide molecules were obtained by polygalacturonase digestion of PGA. It was recently shown that polymyxin B, an antimicrobial peptide that attacks the cell envelope, induces oxidative stress in *E. coli* (Dong et al., 2015). Therefore as a control, we measured ROS production by *Y. enterocolitica* Ye9 and *R. etli* CE3 caused by polymyxin B.

In comparison with the untreated control, sOGAs induced an almost 2-fold increase in ROS level in *R. etli* CE3 cells, but this effect was not observed in the case of *Y. enterocolitica* Ye9 (Figure 9). Conversely, we found no polymyxin B-induced effect on the production of ROS in *R. etli* CE3, but treatment with this antimicrobial agent caused an accumulation of ROS in *Y. enterocolitica* Ye9 (Figure 9). In contrast to polymyxin B, sOGAs appeared to have no effect on the production of ROS in *Y. enterocolitica*.

### Pectin utilization and plant tissue maceration by *Y. enterocolitica* strains

**Figure 10:**
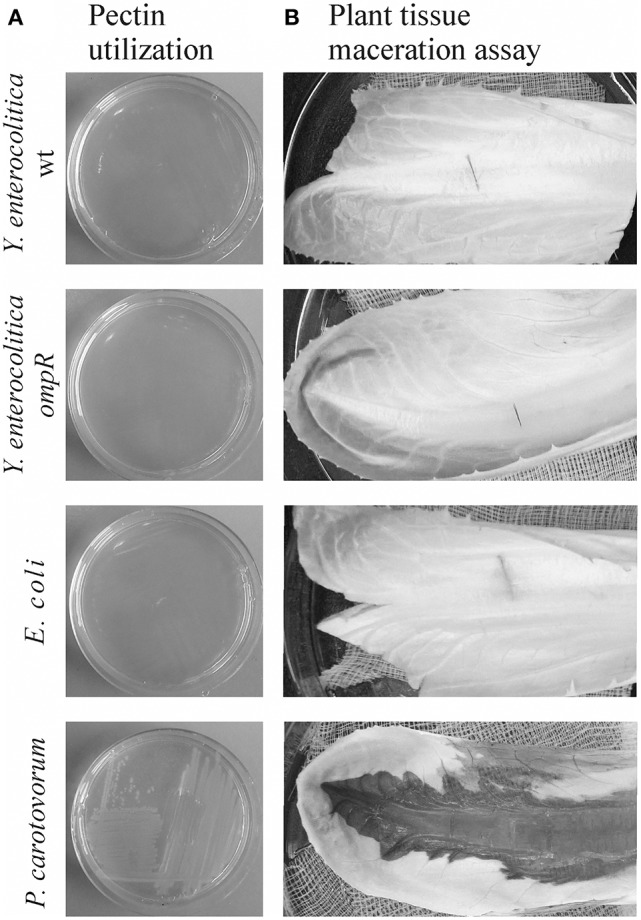
Pectin utilization **(A)** and plant tissue maceration assay **(B)**. **(A)** The ability of *Y. enterocolitica* strains to utilize pectin was assessed visually by analyzing the growth of strains on MMA plates supplemented with 2% (w/v) pectin after incubation at room temperature for 48 h. **(B)** Aliquots of 10 μl of each bacterial suspension (~10^8^ cells) were injected into chicory leaves. The inoculated leaves were incubated in a moist chamber at room temperature for 24 h and then examined for evidence of tissue maceration. The following bacterial strains were tested in this assay: *Y. enterocolitica* wild-type Ye9 and *ompR* mutant AR4, *P. carotovorum* PCM 2056 (positive control) and *E. coli* W (negative control).

Strains of *Y. enterocolitica* differing in their OmpR content were examined for their ability to grow on plates supplemented with 2% pectin (Figure 10A) and macerate chicory leaves (Figure 10B). While *P. carotovorum* PCM 2056 was able to grow on the pectin plates, no visible growth of *Y. enterocolitica* strain Ye9 or the *ompR* mutant AR4 was observed. When *P. carotovorum* PCM 2056 was injected into chicory leaves, a black, macerated lesion developed within 12 h. Neither wild-type Ye9 nor the *ompR* mutant AR4 was capable of leaf tissue maceration in comparison with *P. carotovorum* (Figure 10B). This lack of maceration ability was also observed in *kdgR* and *ompRkdgR* mutants of *Y. enterocolitica* (data not shown). *E. coli*, which lacks OGA transport proteins and pectinases was applied as a negative control and was also incapable of tissue maceration.

### Antimicrobial susceptibility testing

We measured the antibiotic susceptibility of wild-type *Y. enterocolitica* Ye9 and different mutant strains using the broth micro-dilution test. The *ompR* mutant strain AR4 (lacking OmpC and OmpF porins, with strong upregulation of KdgM2) showed reduced sensitivity to the tested β-lactam antibiotics and tetracycline (Table 1). However, the antibiotic susceptibility of the *ompRkdgM2* double mutant strain AR10 was not markedly different from that of the *ompR* mutant. These data implied that the resistant phenotype correlated with the loss of the general porins OmpC and OmpF (confirming our previous observation, Brzostek and Raczkowska, 2007) and that KdgM2 is not a significant entry route for antibiotics. The finding that the null *kdgR* mutation (upregulation of both KdgM1 and KdgM2 porins) did not render *Y. enterocolitica* cells more sensitive to any of the tested antimicrobial compounds supports this conclusion.

**Table 1:** Antibiotic susceptibility of *Y. enterocolitica* strains.

**Antibiotic**	MIC (μg/ml)^*^			
	wild-type (OmpC/F^+^, KdgM1^−^/2^−^^**^)	***kdgR* (OmpC/F^+^, KdgM1^+^/M2^+^)**	***ompR* (OmpC/F^−^, KdgM1^−^/2^+^)**	***ompRkdgM2* (OmpC/F^−^, KdgM1^−^/2^−^)**
Ampicillin	100	100	400	400
Cefotaxime	0.063	0.063	0.125	0.125
Ceftazidime	0.031	0.031	0.25	0.25
Cephaloridine	1.953	1.953	500	500
Cephalothin	15.625	15.625	500	500
Chloramphenicol	3.125	3.125	3.125	3.125
Tetracycline	0.313	0.313	0.625	0.625

* *Three independent replicates gave an identical MIC value*.

** *The production of porins KdgM1 and KdgM2 is inhibited in LB medium*.

### Porin KdgM2 enhances the outer membrane permeability in *Y. enterocolitica*

**Figure 11:**
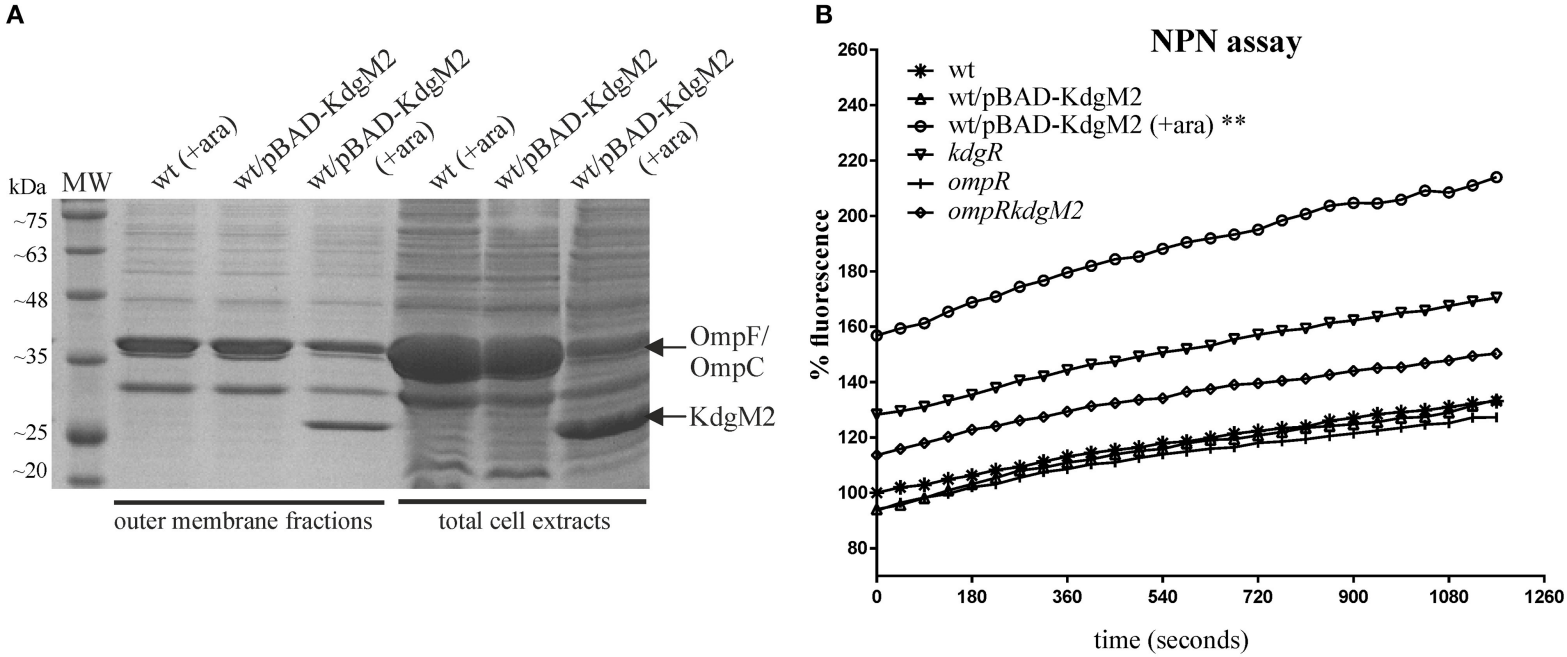
Overexpression of *kdgM2* in *Y. enterocolitica* leads to increased production of KdgM2 in the OM **(A)** and membrane permeability to NPN **(B)**. **(A)** Coomassie blue-stained SDS-PAGE gel shows overproduction of KdgM2 analyzed in OM fractions prepared from bacterial cells grown in LB to exponential phase (OD_600_ ~ 0.5) and in total cell extracts (overnight cultures). Cultures of the wild-type strain Ye9 and Ye9 carrying pBAD-KdgM2 were induced with 0.2% L-arabinose for 1 h (OM fractions) or overnight (total extracts) at 26°C. A non-induced control culture of Ye9 carrying pBAD-KdgM2 was processed in parallel. The KdgM2 protein band is marked. MW – molecular weight standards (3-Color Prestained Protein Marker, DNA-Gdańsk). **(B)** Fluorescent dye NPN uptake by wild-type strain Ye9, strain Ye9/pBAD-KdgM2 induced with L-arabinose to overexpress *kdgM2*, non-induced Ye9/pBAD-KdgM2, *kdgR* mutant ES1 (*kdgR*), *ompR* mutant AR4 (*ompR*) and the *ompRkdgM2* double mutant AR10 (*ompRkdgM2*). Values are averages of three biological replicates. *P-*values were calculated using a one-way ANOVA comparing test strains to the wild-type strain at 19.5 min (^**^P < 0.01).

To address the possibility that the porin KdgM2 can influence outer membrane permeability of *Y. enterocolitica*, an NPN accumulation assay was applied. The use of NPN to study the structure and function of biological membranes is well documented (Hancock, 1984; Loh et al., 1984). NPN, a neutral hydrophobic fluorescent probe, is normally excluded by the outer membrane but exhibits increased fluorescence intensity when it partitions into this membrane. This assay was performed for wild-type strain Ye9, the *kdgR* mutant ES1, the *ompR* mutant AR4 and the *ompRkdgM2* double mutant AR10. Given the influence of OmpR on the outer membrane protein composition, particularly affecting levels of porin KdgM2 (Figure 2A), we also used this assay to examine a strain overexpressing KdgM2. The *kdgM2* coding sequence was cloned into the high-copy-number vector pBAD18Km to generate pBAD-KdgM2 and this was introduced into the wild-type strain Ye9. Induction with L-arabinose led to overexpression of KdgM2 that was detected by SDS-PAGE analysis (Figure 11A). Preliminary experiments were carried out using a growth temperature of 26°C (optimal for *Y. enterocolitica*), but we saw no change in NPN fluorescence in any of the studied strains (data not shown). However, when the assay was performed at 37°C, clearly visible changes were apparent (Figure 11B). The greatest increase in NPN fluorescence was observed in the Ye9 strain overexpressing KdgM2 (^**^P < 0.01). In contrast, we observed no meaningful difference in fluorescence between the wild-type strain, *ompR, kdgR* and double *ompRkdgM2* mutants. These data suggested that overexpression of KdgM2 increases the outer membrane permeability in *Y. enterocolitica*.

### Susceptibility of *Y. enterocolitica* strains to hydrophobic antimicrobial compounds and detergents

The enhanced outer membrane permeability caused by raised levels of KdgM2 (Figure 11), prompted us to perform antimicrobial susceptibility assays using the hydrophobic antibiotic gentamicin and trimethoprim (Delcour, 2009). We examined the level of gentamicin and trimethoprim resistance of the wild-type strain Ye9 and Ye9 overexpressing KdgM2 grown at 26°C and 37°C. L-arabinose induction of pBAD-KdgM2 in the wild-type strain Ye9 grown at 37°C significantly enhanced sensitivity to trimethoprim (MIC 3.125 μg/ml) while this did not occur in the absence of the inducer or in the wild-type strain control, at both temperature (MIC 12.5 μg/ml). The susceptibility to gentamicin was not changed in all studied variants.

The role of porin KdgM2 was further explored using a detergent sensitivity assay. The growth of strains differing in their OmpR, KdgR, and KdgM2 contents was assessed in the presence of two detergents: cationic CTAB and anionic SDS (Zou et al., 2011). The wild-type Ye9 was able to grow in up to 200 SDS μg/ml and 1.56 μg/ml CTAB. In comparison, the *ompR* mutant AR4 displayed only a slight increase in sensitivity to SDS (100 μg/ml), while the overexpression of KdgM2 in the wild-type background did not render the cells more sensitive to either of the tested detergents. These findings indicated that the anionic nature of SDS may be a key determinant of its antimicrobial activity against the *ompR* mutant strain.

## Discussion

Comparative proteomic analysis suggested that the production of *Y. enterocolitica* outer membrane protein KdgM2, related to oligogalacturonide (OGA)-specific porins of *D. dadantii*, might be negatively regulated by OmpR (Nieckarz et al., 2016). The use of SDS-PAGE analysis followed by mass spectrometry in the present study confirmed the strong negative effect exerted by OmpR on the level of KdgM2.

The *kdgM* and *kdgN* genes of *D. dadantii* were shown to be regulated by KdgR, a local repressor of operons/genes involved in pectin catabolism (Nasser et al., 1992; Rodionov et al., 2004). Since the KdgR protein of *Y. enterocolitica* exhibits high sequence similarity (88%) to the corresponding regulatory protein of *D. dadantii*, we reasoned that it should play a similar role in the regulation of *kdgM1* and *kdgM2* expression in *Y. enterocolitica*. Our data showed an increase in the expression of both genes in the *Y. enterocolitica kdgR* deletion mutant, confirming the repression normally exerted by KdgR. In addition, the role of OGAs as an inducer of *kdgM1* and *kdgM2* expression was noted. Induction of OGA-specific porins in the presence of pectin derivatives in *D. dadantii* mainly results from the interaction of the KdgR repressor with an intracellular OGA catabolite such as 2-keto-3-deoxygluconate (KDG) (Hugouvieux-Cotte-Pattat et al., 1996). Since *Y. enterocolitica* can grow on OGAs as the sole carbon source, we speculate that OGA catabolism involves a stage in which KDG is formed. An *in silico* search of the regulatory regions of the *Y. enterocolitica kdgM1* and *kdgM2* genes using the consensus KdgR binding site sequence of *D. dadantii* identified highly similar sequence elements.

The results of reporter gene fusion assays demonstrated that the expression of *kdgM2* is inhibited by OmpR. The additive effect of *kdgR* and *ompR* mutations revealed that KdgR and OmpR regulate *kdgM2* expression independently. Analysis of *kdgM1* expression suggested the positive role of OmpR irrespective of the presence of KdgR. Thus, the expression of *kdgM1* and *kdgM2* appear to be regulated in an inverse manner by OmpR: the former is activated while the latter is repressed. It was previously shown that the production of KdgM and KdgN in *D. dadantii* is also subject to inverse regulation by OmpR, but direct involvement of this regulator in this process has not been reported (Condemine and Ghazi, 2007). In addition, OmpR of *D. dadantii* was shown to repress the expression of *kdgM* located downstream of the *pelW-togMNAB* operon, but we found that OmpR of *Y. enterocolitica* produces the opposite regulatory effect (upregulation) on the homolog *kdgM1*, located in the same genetic context. OmpR has the reciprocal effect on the expression of *kdgN* of *D. dadantii*, i.e., activation, whereas its *Y. enterocolitica* homolog *kdgM2* is repressed by this regulator.

We found that the expression of both *kdgM* genes are inhibited by high osmolarity, although in an OmpR-independent manner. It may be speculated that other regulatory factors or mechanisms might contribute to the modulation of *kdgM1* and *kdgM2* transcription in response to varying osmolarity, as has been reported for some enterobacterial genes (Higgins et al., 1988). Interestingly, some genes regulated by EnvZ/OmpR are not sensitive to osmotic change. The *tpp* genes from *Salmonella enterica* sv Typhimurium and *E. coli* are notable examples (Gibson et al., 1987; Goh et al., 2004). Osmoregulation of *kdgN* expression in *D. dadantii* has been reported previously, and a marginal role for OmpR in this phenomenon was proposed (Condemine and Ghazi, 2007).

In search of other OmpR-regulated genes of the pectinolytic pathway, we performed a bioinformatic analysis to identify putative OmpR-binding sites within regulatory regions. Unexpectedly, the *in silico* analysis of the 5′ untranslated region of *kdgR* revealed the presence of two putative OmpR-binding sequences. Reporter gene fusion assays and RT-qPCR demonstrated that the expression of KdgR is negatively regulated by OmpR.

Besides the *Y. enterocolitica kdgR* gene, putative OmpR-binding sites were detected in the promoter regions of the *kdgM2-pelP-sghX* operon (1 site) and *kdgM1* gene (2 sites). In addition, single putative KdgR-binding sites were identified in the promoter regions of the *kdgM2-pelP-sghX* operon and *kdgM1* gene. The OmpR- and KdgR-binding sites in the *kdgM2* promoter region are separated by 63 bp. In the *kdgM1* regulatory region, one of the two putative OmpR-binding sites overlaps the KdgR-binding site, suggesting that OmpR bound at this site could act as an antirepressor by preventing KdgR binding. However, the results of reporter gene fusion assays demonstrated a positive role for OmpR in *kdgM1* expression and indicated that OmpR and KdgR act independently to regulate this gene. EMSAs confirmed the binding of *Y. enterocolitica* OmpR to regulatory region fragments of the *kdgM2-pelP-sghX* operon, and the *kdgM1* and *kdgR* genes. This is strong evidence of a direct role for OmpR in the regulation of KdgM2, KdgM1, and KdgR biosynthesis.

The production of porins KdgM1 and KdgM2 is likely to be influenced directly and indirectly by OmpR. The positive regulation of *kdgM1* by OmpR may result from both its direct positive effect on *kdgM1* expression and the indirect effect of its inhibition of *kdgR* expression. The observed strong negative regulation of *kdgM2* by OmpR suggests that the direct inhibitory effect it exerts on this gene is more powerful than any upregulation caused by OmpR-dependent inhibition of *kdgR* expression. However, we cannot rule out the possibility that additional, as yet uncharacterized regulators might exist that are involved in the regulation of *kdgM1* and *kdgM2* in *Y. enterocolitica*. The transcriptional regulatory network controlling the *kdgM* and *kdgN* genes of *D. dadantii* is highly complex and composed of several regulatory proteins, i.e., KdgR, PecS, HNS, OmpR and CRP as well as their cross-regulatory interactions (Blot et al., 2002; Condemine and Ghazi, 2007; Sepulchre et al., 2007). Interestingly, no elements with sequence homology to the consensus OmpR-binding site were detected in the regulatory regions of the *D. dadantii kdgM* or *kdgN* genes (Condemine and Ghazi, 2007).

**Figure 12:**
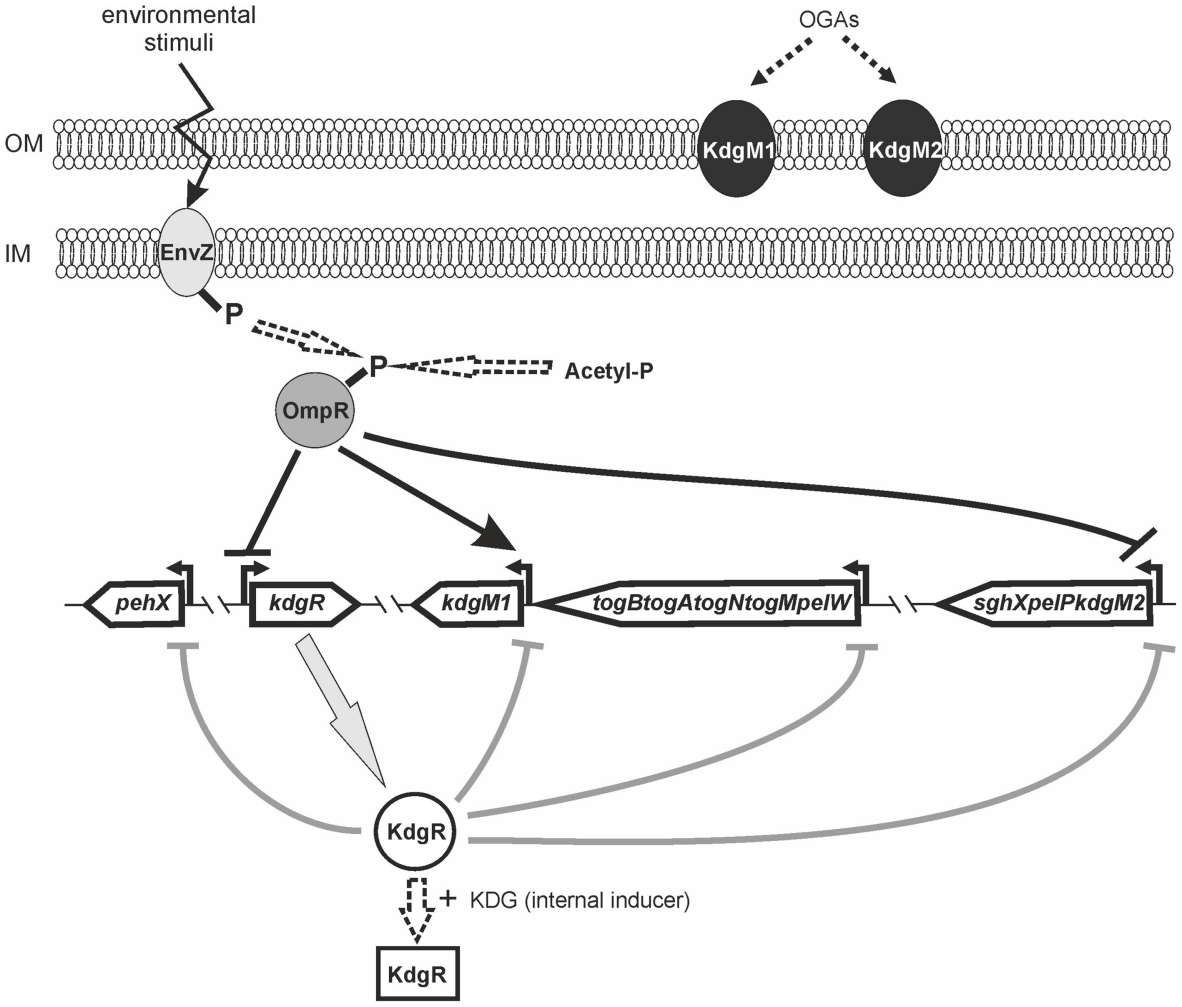
Model of the OmpR and KdgR regulatory network controlling gene expression involved in the uptake and depolymerization of oligogalacturonides in *Y. enterocolitica*. Oligogalacturonides (OGAs) transported from the environment into *Y. enterocolitica* cells via the KdgM porins can be used as a carbon source. OGA uptake and catabolism are controlled at the transcriptional level by repressor KdgR. Binding of the OGA degradation product 2-keto-3-deoxygluconate (KDG) by KdgR leads to its inactivation, resulting in derepression of genes/operons of the KdgR regulon. The transcriptional regulation of *kdgM1, pehX, pelW-togMNAB*, and *kdgM2-pelP-sghX* expression is mediated by KdgR and OmpR regulators. OmpR activated (phosphorylated) by an unidentified environmental signal directly and/or indirectly regulates expression of the genes/operons of KdgR regulon. OmpR-P binding to the promoter regions of *kdgM1* and *kdgM2-pelP-sghX* leads to their activation and repression, respectively. Binding of OmpR-P to the *kdgR* regulatory region inhibits its transcription causing indirect upregulation of *kdgM1* and *kdgM2-pelP-sghX*. The expression of *pehX* and *pelW-togMNAB* is indirectly upregulated by OmpR-P. The different regulatory effects at the transcriptional level are indicated by an arrow for positive regulation or by a line with a bar for negative regulation. The possible roles of EnvZ and acetyl-phosphate in OmpR phosphorylation as well as KDG in the inactivation of KdgR are denoted by dotted arrows.

If OmpR negatively regulates *kdgR*, other genes of the *Y. enterocolitica* KdgR regulon should be indirectly influenced by OmpR. Using reporter gene fusion assays we found that the *pehX* gene, encoding the polygalacturonase PehX and the *pelW-togMNAB* operon, encoding the cytoplasmic exopolygalacturonate lyase PelW and oligogalacturonide transport system TogMNAB are positively regulated by OmpR. Both transcriptional units are members of the KdgR regulon. It is worth nothing that while we identified KdgR binding motifs in the respective promoter regions, we were unable to detect appropriate OmpR-binding sites. Thus, the regulatory effect of OmpR on *pehX* and *pelW-togMNAB* may be linked to its influence on *kdgR* expression. Together, our findings show that the effects of OmpR on some members of the KdgR regulon are likely to be direct, by transcriptional control of particular genes, and/or indirect, by modulation of the expression of other regulatory factor genes, including *kdgR* (see model, Figure 12.)

In the course of this study we verified the hypothesis that an OM channel formed by *Y. enterocolitica* KdgM2 might represent a route via which low molecular weight hydrophilic β-lactam antibiotics can enter the cell. However, neither upregulation of KdgM2 in the absence of the general porins OmpC and OmpF in the *ompR* mutant nor the upregulation of both KdgM1 and KdgM2 in the *kdgR* mutant, affected the antibiotic sensitivity of these strains, which argues against a role for these oligogalacturonide-specific porins in the penetration of the studied drugs. Interestingly, specific porins like maltoporin LamB and phosphate transport porin PhoE have been shown to contribute to resistance to certain β-lactam antibiotics in *Klebsiella pneumoniae* (Kaczmarek et al., 2006; Garcia-Sureda et al., 2011).

Interestingly, overexpression of KdgM2 in *Y. enterocolitica* was found to (i) increase outer membrane permeability, as revealed by the accumulation of hydrophobic dye NPN, and (ii) make cells more susceptible to the trimethoprim. These results corroborate the recent finding in *Salmonella* that an increase in KdgM proteins in a mutant strain lacking elongation factor P leads to increased OM permeability (Zou et al., 2011). However, contrary to the *Salmonella* data we were unable to demonstrate increased susceptibility to the gentamicin, probably due to the presence of a functional AcrAB efflux system in *Y. enterocolitica* (Raczkowska et al., 2015). Interestingly, the increased permeability of *Y. enterocolitica* membranes caused by overexpressed KdgM2 was only observed at 37°C and not at the normal growth temperature of 26°C. Notably, this raised temperature is also known to inhibit the synthesis of the O-polysacharide chain of lipopolysaccharide (LPS) in yersiniae cells (Bengoechea et al., 2004; Skurnik et al., 2007). Thus, the biological relevance of KdgM2 may be associated with growth of *Y. enterocolitica* at 37°C, i.e., the body temperature of a mammalian host. The tight negative regulation of *kdgM2* expression by OmpR would therefore be necessary to decrease OM permeability and prevent the influx of deleterious antimicrobial factors produced by the host.

Our data raised questions concerning the redundancy of KdgM porins in *Y. enterocolitica* and the adaptive role of OmpR associated with the modulation of their levels. The differential regulation of KdgM1 and KdgM2 by OmpR might reflect the varied function of these proteins in bacteria growing in different environmental niches, as has been proposed for OmpC and OmpF in *E. coli* (Nikaido, 2003). *Y. enterocolitica* exhibits a dual lifestyle, existing as both a non-pathogenic saprophyte and a pathogen residing inside the host body. These two environments differ greatly in the nature of the carbon and energy sources available and in the presence and concentration of harmful compounds. In the saprophytic lifestyle, *Y. enterocolitica* may utilize both KdgM porins to acquire OGAs from the surrounding environment. In the host body, upregulation of more specific channels may be necessary for the uptake of OGAs present in the intestinal environment (Cummings et al., 1979; Cummings and Englyst, 1987; Gibson et al., 1990). OGAs could be derived from pectin degradation mediated by the pectinolytic activity of symbiotic microbiota sharing the same ecological niche. On the other hand, the inhibition of KdgM2 production by OmpR might decrease OM permeability and limit the diffusion of harmful compounds into cells growing within a mammalian host. Thus, production of the appropriate KdgM porin for a particular local environment, mediated by the regulator OmpR, might contribute to the fitness of *Y. enterocolitica*.

## Author contributions

MN designed and performed experiments, analyzed data and helped to write the paper. AR performed some experiments and analyzed data. KJ, ES, KS, and DS helped to perform some experiments. KB designed the experiments, analyzed data, wrote the paper and provided financial support.

### Conflict of interest statement

The authors declare that the research was conducted in the absence of any commercial or financial relationships that could be construed as a potential conflict of interest.

## Supplementary material

Figure S1 Amino acid sequence alignment of selected KdgM family members from *Yersiniae, D. dadantii*, and *P. carotovorum*. **(A)** The aligned amino acid sequences are KdgM1 and KdgM2 from *Y. enterocolitica* subsp. *palearctica* 105.5R(r) (Y.ent.; Taxonomy ID: 994476), *Y. pseudotuberculosis* IP 32953 (Y.pse.; Taxonomy ID: 273123), *Y. pestis* KIM10+ (Y.pes.; Taxonomy ID: 187410), KdgN and KdgM from *D. dadantii* 3937 (D.dad.; Taxonomy ID: 198628), and KdgN, KdgM, KdgM3, and KdgM4 from *P. carotovorum* subsp. *carotovorum* PC1 (P.car.; Taxonomy ID: 561230). Identical residues are marked by asterisks, highly similar residues are denoted by colons and slightly similar residues are indicated by periods. The program T-Coffee (version 8.93) was used to draw the alignment and the sequence accession numbers are shown. **(B)** Percentage identity between KdgM1/KdgM2 from *Y. enterocolitica* subsp. *palearctica* 105.5R(r) and the other KdgM proteins determined using Protein BLAST.

Figure S2 Alignment of the KdgR amino acid sequence of *Y. enterocolitica* subsp. *palearctica* 105.5R(r) (Y.ent.O:9; Acc.no. ADZ42452) with those of *D. dadantii* 3937 (D.dad.;Acc. no. ADM98638) and *P. carotovorum* subsp. *carotovorum* PC1 (P.car.; Acc. no. ACT12926). Identical residues are marked by asterisks, highly similar residues are denoted by colons and slightly similar residues are indicated by periods. The HTH motif is boxed in gray. The program T-Coffee (version 8.93) was used to draw the alignment.

Table S1 Strains and plasmids used in this study.

Table S2 Oligonucleotide primers used in this study.

Additional File 1 Bioinformatic analysis of *Y. enterocolitica* proteins of the KdgM family.
