# Supplementary figures and images for: The Role of OmpR in the Expression of Genes of the KdgR Regulon Involved in the Uptake and Depolymerization of Oligogalacturonides in Yersinia enterocolitica
Source: Front Cell Infect Microbiol. 2017 Aug 15;7:366. doi: 10.3389/fcimb.2017.00366 (PMC5559549; doi:10.3389/fcimb.2017.00366)

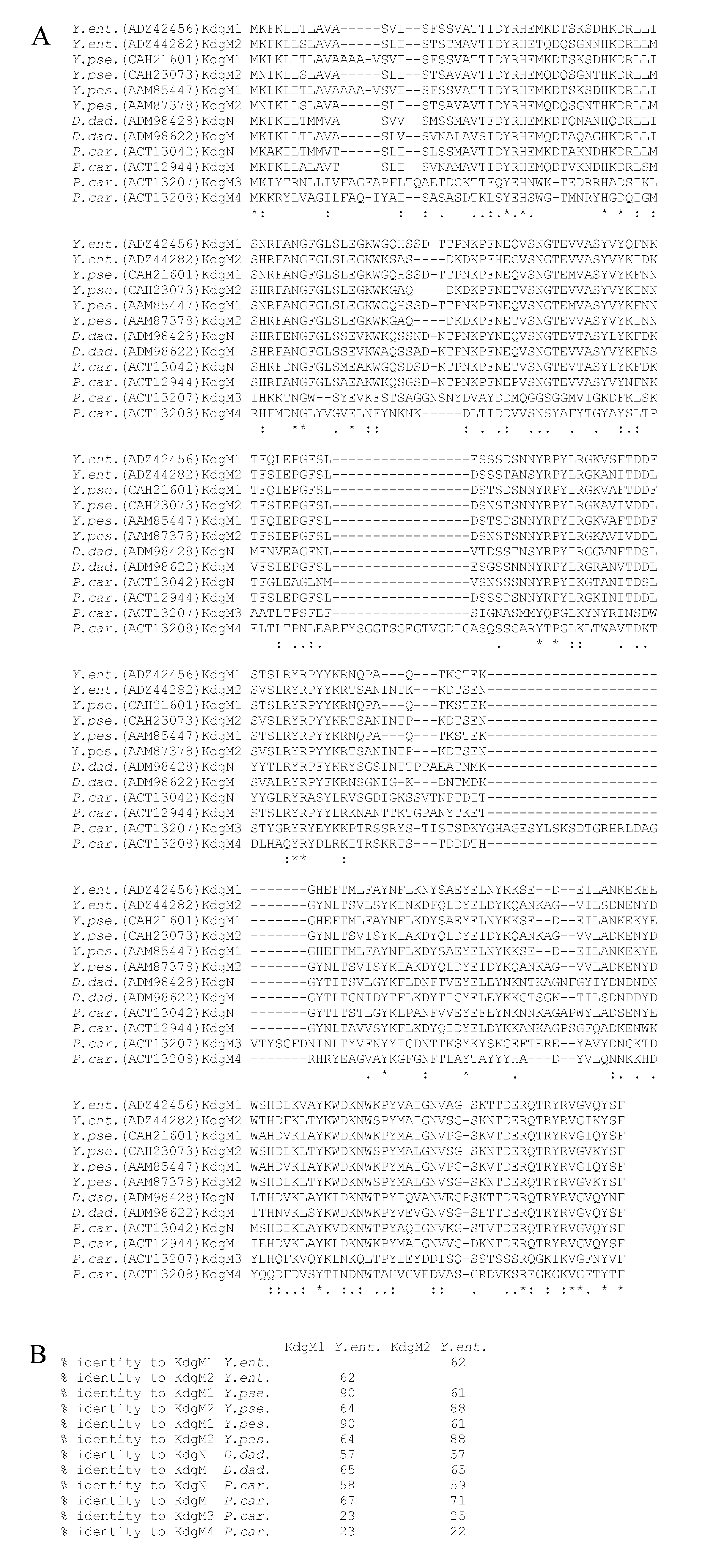

Supplement: Figure S1 — Amino acid sequence alignment of selected KdgM family members from Yersiniae, D. dadantii, and P. carotovorum. (A) The aligned amino acid sequences are KdgM1 and KdgM2 from Y. enterocolitica subsp. palearctica 105.5R(r) (Y.ent.; Taxonomy ID: 994476), Y. pseudotuberculosis IP 32953 (Y.pse.; Taxonomy ID: 273123), Y. pestis KIM10+ (Y.pes.; Taxonomy ID: 187410), KdgN and KdgM from D. dadantii 3937 (D.dad.; Taxonomy ID: 198628), and KdgN, KdgM, KdgM3, and KdgM4 from P. carotovorum subsp. carotovorum PC1 (P.car.; Taxonomy ID: 561230). Identical residues are marked by asterisks, highly similar residues are denoted by colons and slightly similar residues are indicated by periods. The program T-Coffee (version 8.93) was used to draw the alignment and the sequence accession numbers are shown. (B) Percentage identity between KdgM1/KdgM2 from Y. enterocolitica subsp. palearctica 105.5R(r) and the other KdgM proteins determined using Protein BLAST. [file Image1.TIF]

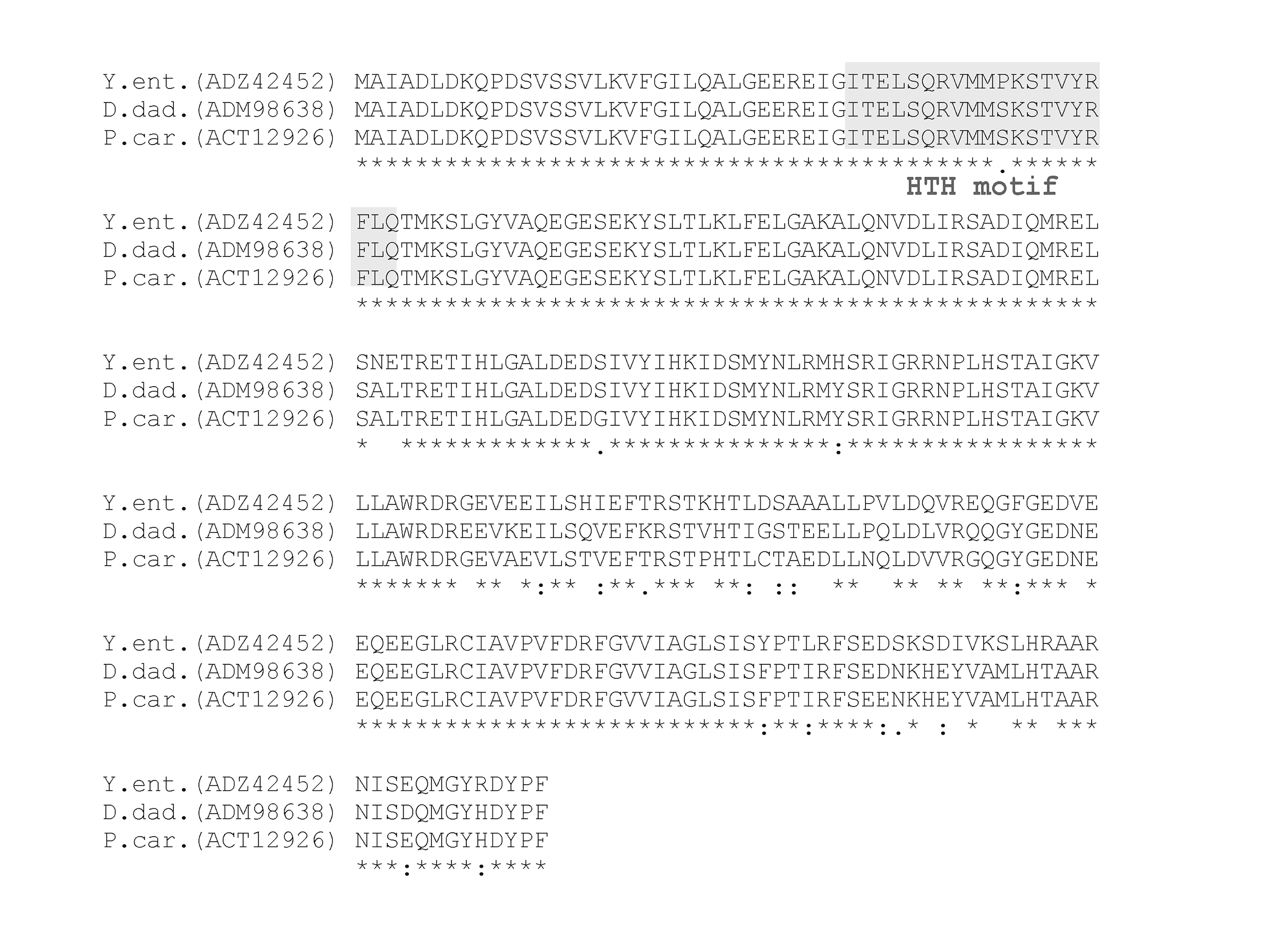

Supplement: Figure S2 — Alignment of the KdgR amino acid sequence of Y. enterocolitica subsp. palearctica 105.5R(r) (Y.ent.O:9; Acc.no. ADZ42452) with those of D. dadantii 3937 (D.dad.;Acc. no. ADM98638) and P. carotovorum subsp. carotovorum PC1 (P.car.; Acc. no. ACT12926). Identical residues are marked by asterisks, highly similar residues are denoted by colons and slightly similar residues are indicated by periods. The HTH motif is boxed in gray. The program T-Coffee (version 8.93) was used to draw the alignment. [file Image2.TIF]
