# Supplementary material for: The Role of OmpR in the Expression of Genes of the KdgR Regulon Involved in the Uptake and Depolymerization of Oligogalacturonides in Yersinia enterocolitica
Source: Front Cell Infect Microbiol. 2017 Aug 15;7:366. doi: 10.3389/fcimb.2017.00366 (PMC5559549; doi:10.3389/fcimb.2017.00366)
Supplement: Table S1 — Strains and plasmids used in this study. [file Table1.DOCX]

**Table S1. Strains and plasmids used in this study**.

| **Strains and plasmids** | **Description** | **Reference or source** |
| --- | --- | --- |
| ***Y. enterocolitica* O:9** | | |
| Ye9 | wild-type, clinical isolate of serotype O:9, carrying virulence plasmid pYV | Clinical isolate, laboratory collection |
| Ye9N | Ye9 derivative, spontaneous Nal^R^ mutant | Brzostek et al., 2007 |
| Ye9NK1 | Ye9N derivative carrying a *kdgM1::lacZYA’* fusion, Nal^R^, Cm^R^ | This work |
| ES1 | Ye9N derivative, Δ*kdgR*::Gm defective in KdgR production, Nal^R^, Gm^R^ | This work |
| ES1K1 | ES1 derivative carrying a *kdgM1::lacZYA’* fusion, Nal^R^, Gm^R^, Cm^R^ | This work |
| MN1 | Ye9N derivative, Δ*kdgM2*::Gm defective in KdgM2 production, Nal^R^, Gm^R^ | This work |
| AR4 | Ye9N derivative Δ*ompR*::Km defective in OmpR production, Nal^R^, Km^R^ | Brzostek et al., 2003 |
| AR4K1 | AR4 derivative carrying a *kdgM1::lacZYA’* fusion, Nal^R^, Km^R^, Cm^R^ | This work |
| AR11 | AR4 derivative Δ*kdgR*::Gm defective in KdgR production, Nal^R^, Km^R^, Gm^R^ | This work |
| AR11K1 | AR11 derivative carrying a *kdgM1::lacZYA’* fusion, Nal^R^, Km^R^, Gm^R^, Cm^R^ | This work |
| AR10 | AR4 derivative, Δ*kdgM2*::Gm defective in KdgM2 production, Nal^R^, Km^R^, Gm^R^ | This work |
| ***E. coli*** | | |
| S17-1 λpir |  | Simon et al., 1983 |
| TOP10 F’ |  | Invitrogen |
| DH5α |  | Sambrook et al., 1989 |
| BL21 (DE3) |  | Life Technologies |
| TG1 |  | Sambrook et al., 1989 |
| W | ATTC 9637 | ATTC Collection |
| **Other strains** | | |
| *Pectobacterium carotovorum* subsp. *carotovorum* PCM 2056 | wild-type strain | PCM Collection* |
| *Rhizobium etli* CE3 | spontaneous Sm^R^ derivative of wild-type strain CFN42 | Noel et al., 1984 |
| PLASMIDS | | |
| pDrive | cloning vector, Ap^R^, Km^R^ | Qiagen |
| pFUSE | suicide vector, derivative of pEP185.2 with promoterless *lacZYA* genes, Cm^R^ | Baumler et al., 1996 |
| pFkdgM1 | pFUSE with XbaI/SmaI fragment (549-bp) of *kdgM1*, Cm^R^ | This work |
| pBluescript II SK(+) | *ori* M13, ori pBR322, general cloning vector, Ap^R^ | Stratagene |
| pBluescript/ *kdgM2’-‘rfp* | pBluescript II SK (+) carrying 264 bp upstream of the *kdgM2* start codon and 10 codons of *kdgM2* fused in frame with *rfp*, Ap^R^ | This work |
| pBBR1MCS-5 | broad-host-range cloning vector, *ori* pBBR1, Mob^+^, *oriT* RK2, Gm^R^ | Kovach et al*.*, 1995 |
| pBKRFP | pBBR1MCS-5 derivative carrying *kdgM2’-‘rfp*  cloned into BamHI and EcoRI sites | This work |
| p34E-Tp | source of trimethoprim cassette, Ap^R^,Tp^R^ | Deshazer and Woods, 1996 |
| pBKRFP-Tp | pBKRFP derivative carrying Tp^R^ cassette from p34E-Tp inserted into the SalI site | This work |
| pDS132 | *ori* R6K (narrow host range, replication only in *E. coli* λpir), *oriT* RK2, *sacB*, Cm^R^ | Philippe  et al., 2004 |
| pDSkdgR | pDS132 derivative carrying 2196-bp cassette for *kdgR* mutagenesis constructed by overlap extension PCR cloned between XbaI sites of the vector, Gm^R^ | This work |
| pDSkdgM2 | pDS132 derivative carrying 2186-bp cassette for *kdgM2* mutagenesis constructed by overlap extension PCR cloned between XbaI sites of the vector, Gm^R^ | This work |
| pCM132Gm | pCM132 derivative, ori pMB1, ori RK2, oriT RK2, promoterless *lacZ* gene, Gm^R^ | DBG collection** |
| pCM132Gm-*kdgR::lacZ* | pCM132Gm derivative carrying 479 bp upstream of *kdgR* start codon and 95 bp of ORF *kdgR* cloned upstream of promoterless *lacZ* gene between EcoRI and KpnI sites | This work |
| pCM132Gm-*pehX::lacZ* | pCM132Gm derivative carrying 628 bp upstream of *pehX* start codon and 77 bp of ORF *pehX* cloned upstream of promoterless *lacZ* gene between EcoRI and KpnI sites | This work |
| pCM132Gm-*pelW-togMNAB::lacZ* | pCM132Gm derivative carrying 594 bp upstream of *pelW* start codon and 108 bp of ORF *pelW* cloned upstream of promoterless *lacZ* gene between EcoRI and KpnI sites | This work |
| pETOmpR | pET28a carrying the entire *ompR* coding sequence (725-bp fragment), Km^R^ | Nieckarz et al., 2016 |
| pHR4 | pHSG575 with 740-bp fragment of *ompR* (ORF with rbs), Cm^R^ | Brzostek et al., 2003 |
| pBR3 | pBBR1MCS-3 with XhoI/PstI fragment  containing entire coding sequence of *ompR* (ORF with rbs), Tet^R^ | Brzostek et al., 2007 |
| pHSG575 | low copy number cloning vector, Cm^R^ | Takeshita et al., 1987 |
| pkdgR-Cm | pHSG575 with BamHI/HindIII fragment containing entire coding sequence of *kdgR* (ORF with rbs), Cm^R^ | This work |
| pBBR1MCS-3 | broad-host-range cloning vector, *ori* pBBR1, Mob^+^, *oriT* RK2, Tet^R^ | Kovach et al*.*, 1995 |
| pkdgR-Tet | pBBR1MCS-3 with KpnI/SacI fragment containing entire coding sequence of *kdgR* (ORF with rbs), Tet^R^ | This work |
| pBAD18Km | Arabinose-regulated expression plasmid, Km^R^ | Guzman et al., 1995 |
| pBAD-kdgM2 | pBAD18Km with SacI/SphI fragment containing entire coding sequence of *kdgM2* (ORF with rbs), Km^R^, *kdgM2* under the control of the inducible pBAD promoter | This work |
| pRK2013 | helper plasmid used to mobilize vectors in triparental mating, Km^R^ | Ditta et al., 1980 |
| *PCM Collection, Polish Collection of Microorganisms - Ludwik Hirszfeld Institute of Immunology and Experimental Therapy, Polish Academy of Sciences (ECCO) | | |
| **Department of Bacterial Genetics*,* Institute of Microbiology, Faculty of Biology, University of Warsaw | | |
| Cm^R^ chloramphenicol resistance, Gm^R^ gentamicin resistance, Km^R^ kanamycin resistance, Nal^R^ nalidixic acid resistance, Sm^R^ streptomycin resistance, Tet^R^ tetracycline resistance, ::Km, insertion of kanamycin resistance cassette; ::Gm, insertion of gentamicin resistance cassette | | |

**REFERENCES**

Ditta, G., Stanfield, S., Corbin, D., Helinski, D.R. (1980). Broad host range DNA cloning system for gram-negative bacteria: construction of a gene bank of Rhizobium meliloti. *Proc. Natl. Acad.* *Sci.* U.S.A. 77, 7347–7351.

Brzostek, K., Raczkowska, A., and Zasada, A. (2003). The osmotic regulator OmpR is involved in the response of *Yersinia enterocolitica* O:9 to environmental stresses and survival within macrophages. *FEMS Microbiol Lett*. 228, 265–271.

Brzostek, K., Brzóstkowska, M., Bukowska, I., Karwicka, E., and Raczkowska, A. (2007). OmpR negatively regulates expression of invasin in *Yersinia enterocolitica. Microbiol*. 153, 2416–2425. doi: 10.1099/mic.0.2006/003202-0

Deshazer, D., and Woods, D.E. (1996). Broad-host-range cloning and cassette vectors based on the R388 trimethoprim resistance gene. *Biotechniques*. 20, 762–764.

Guzman, L.M., Belin, D., Carson, M.J., and Beckwith J. (1995). Tight regulation, modulation, and high-level expression by vectors containing the arabinose PBAD promoter*. J. Bacteriol.* 177, 4121–4130.

Kovach, M.E., Elzer, P.H., Hill, D.S., Robertson, G.T., Farris, M.A., Roop, R.M., and Peterson, K.M. (1995). Four new derivatives of the broad-host-range cloning vector pBBR1MCS, carrying different antibiotic-resistance cassettes. *Gene*. 166, 175–176.

Nieckarz, M., Raczkowska, A., Dębski, J., Kistowski, M., Dadlez, M., Heesemann, J., et al. (2016). Impact of OmpR on the membrane proteome of Yersinia enterocolitica in different environments: repression of major adhesin YadA and heme receptor HemR. *Environ*. *Microbiol*. 18, 997–1021, doi: 10.1111/1462-2920.13165.

Noel, K.D., Sanchez, A., Fernandez, L., Leemans, J., and Cevallos, M.A. (1984). *Rhizobium phaseoli* symbiotic mutants with transposon Tn5 insertions. *J. Bacteriol.* 158, 148–155.

Philippe, N., Alcaraz, J.P., Coursange, E., Geiselmann, J., and Schneider. D. (2004). Improvement of pCVD442, a suicide plasmid for gene allele exchange in bacteria. *Plasmid.* 51, 246–255. doi: 10.1016/j.plasmid.2004.02.003

Sambrook, J., Fritsch, E.F., and Maniatis, T. (1989). Molecular Cloning: a Laboratory Manual. 2nd ed. Cold Spring Habor, NY, USA: Cold Spring Harbor Laboratory Press.

Simon, R., Priefer, U., and Pühler, A. (1983). A broad host range mobilization system for *in vivo* genetic engineering: transposon mutagenesis in Gram negative bacteria. *Nat. Biotechnol.* 1, 784–791.

Takeshita, S., Sato, M., Toba, M., Masahashi, W. and Hashimoto-Gotoh, T. (1987). High-copy-number and low-copy-number plasmid vectors for *lacZ* alpha-complementation and chloramphenicol- or kanamycin-resistance selection. *Gene.* 61, 63-74.
