# Supplementary material for: The Role of OmpR in the Expression of Genes of the KdgR Regulon Involved in the Uptake and Depolymerization of Oligogalacturonides in Yersinia enterocolitica
Source: Front Cell Infect Microbiol. 2017 Aug 15;7:366. doi: 10.3389/fcimb.2017.00366 (PMC5559549; doi:10.3389/fcimb.2017.00366)
Supplement: Table S2 — Oligonucleotide primers used in this study. [file Table2.DOCX]

**Table S2. Oligonucleotide primers used in this study.**

| **Purpose and Target** | **Name of primer** | **Primer sequence (5’→ 3’)*** | **Restriction enzyme** | **Reference** |
| --- | --- | --- | --- | --- |
| RT-PCR *kdgM2-pelP* | RTkdgMpelP1 | AAAGCGCCAGTGACAAAGAT |  | This study |
|  | RTkdgMpelP2 | ATCAACAAACCGGTGTCCTC |  | This study |
| RT-PCR *pelP-sghX* | RTpelPsghX1 | CCTGGCCGATCAGTATGTTT |  | This study |
|  | RTpelPsghX2 | TTTCCAGACCTTCAGCCAAC |  | This study |
| EMSA *kdgM1* | kdgM1x | **TGTCTAGA**ATCCTTCTGTTGCCGGTTTA | XbaI | This study |
|  | kdgM1s | **TGCCCGGG**TTACAGATGCCACTGCCAGA | SmaI | This study |
| EMSA *kdgM2* | k2M1 | **ACTCTAGA**TGTGTTAGCTTCCCTCACTGG | XbaI | This study |
|  | k2M500 | **ACCCCGGG**AGTTCCGAGATCCATGACTA | SmaI | This study |
| EMSA *kdgR* | kdgReA | CACGACACCTCATGGAAGG |  | This study |
|  | kdgReB | ACCTATTTCCCGTTCTTCACCC |  | This study |
| Negative control for EMSA 16S rDNA | 16SR1 | ATTCCGATTAACGCTTGCAC |  | Nieckarz et al., 2016 |
|  | 16SR304 | GTGGGGTAATGGCTCACCTA |  |  |
| Construction of *kdgR* mutants | KdgR1 | **GCTCTAGA**ATGCTCGCATGTGGCTAATC | XbaI | This study |
|  | KdgR2 | CATCCGTTTCCACGGGTTACCCTACTCA |  | This study |
|  | KdgR3 | TGAGTAGGGTAACCCGTGGAAACGGATG |  | This study |
|  | KdgR4 | AAGATCCCTCAGAACGATCTCGGCTTGA |  | This study |
|  | KdgR5 | TCAAGCCGAGATCGTTCTGAGGGATCTT |  | This study |
|  | KdgR6 | **CGTCTAGA**GTAACATCCGCTCAGTGAA | XbaI | This study |
|  | KdgR0 | TGGGTGCCGTTGACGGATTG |  | This study |
|  | KdgR7 | GGCCAGGGTCAGACTTTCTC |  | This study |
| Construction of *kdgM2* mutants | KdgM1 | **GCTCTAGA**CCACTTCGCCATACTTTGGT | XbaI | This study |
|  | KdgM2 | CTTCATCCGTTTCCACGCATAGCTGGCAACCACT |  | This study |
|  | KdgM3 | AGTGGTTGCCAGCTATGCGTGGAAACGGATGAAG |  | This study |
|  | KdgM4 | GCTGCCGGAAACATTACCGATCTCGGCTTGAACG |  | This study |
|  | KdgM5 | CGTTCAAGCCGAGATCGGTAATGTTTCCGGCAGC |  | This study |
|  | KdgM6 | **CGTCTAGA**GGGCATTCCAAAAACCACGAAT | XbaI | This study |
|  | KdgM0 | AATCACTGGGCTTTAGTCGAA |  | This study |
|  | KdgM7 | ATCGGTTTGCCATATTCACC |  | This study |
| Confirmation the correctness of *kdgM2’-‘rfp* fusion. | LL1 | ATTTAATTCGAAGGCGATCC |  | This study |
|  | RR4 | CGAAGGTGAGCCAGTGTGAC |  | This study |
| Construction of *kdgR::lacZ* transcriptional fusion | KdgREcoRI | **TAGAATTC**ATGATGGTTCGTTGATGGTG | EcoRI | This study |
|  | KdgRKpnI | **TAGGTACC**TTCCCGTTCTTCACCCAAT | KpnI | This study |
| Construction of *pelW-togMNAB::lacZ* transcriptional fusion | PelWEcoRI | **TAGAATTC**GCTGTCATGGGTGTAACTCGT | EcoRI | This study |
|  | PelWKpnI | **TAGGTACC**GCTGTGGCTTACACACTGGA | KpnI | This study |
| Construction of *pehX::lacZ* transcriptional fusion | PehXEcoRI | **TAGAATTC**AGAAAAAGAGTGGCGTCTCG | EcoRI | This study |
|  | PehXKpnI | **TAGGTACC**ATCGGAGTACCCACCATCAG | KpnI | This study |
| Confirmation the correctness of fusions constructed in pCM132Gm | pCM132GmSPR1 | CTGCAAGGCGATTAAGTTGG |  | This study |
|  | pCM132GmSPR2 | CATAAACTGCCAGGCATCAA |  | This study |
| Construction of *kdgM1::lacZ* transcriptional fusion | KdgM1X | **TGTCTAGA**ATCCTTCTGTTGCCGGTTTA | XbaI | This study |
|  | KdgM1S | **TGCCCGGG**TTACAGATGCCACTGCCAGA | SmaI | This study |
| Confirmation the correctness of fusion constructed using pFUSE | LPkdgM2683 | CACAGATGCTTTCCATTGGT |  | This study |
|  | lacZH991 | CATCGCAGGCTTCTGCTTC |  | This study |
| Control of contamination with genomic DNA | Y1 | AATACCGCATAACGTCTTCG |  | Wannet et al., 2001 |
|  | Y2 | CTTCTTCTGCGAGTAACGTC |  |  |
| Construction of pkdgR-Cm | KdgRorfBamHI | **TAGGATCC**TGAAACACAGAAAACTGAGTAGGG | BamHI | This study |
|  | KdgRorfHindIII | **TAAAAGCTT**CCCTAGTGGAAAGATCCCTCA | HindIII | This study |
| Construction of pkdgR-Tet | KdgRorfKpnI | **TAAGGTACC**TGAAACACAGAAAACTGAGTAGGG | KpnI | This study |
|  | KdgRorfSacI | **TAGAGCTC**CCCTAGTGGAAAGATCCCTCA | SacI | This study |
| Construction of pBAD-KdgM2 | ARAkdgM2SacI | **TGAGCTC**GTTATTTATTAAAATAAGGTAACTGTAATG | SacI | This study |
|  | ARAkdgM2SphI | **TATAAGCATGC**TAGCGAGGTGTGACCCCTAA | SphI | This study |
| RT-qPCR | 16SrRNA-F | CACACTGGAACTGAGACA |  | This study |
|  | 16SrRNA-R | TGCTTCTTCTGCGAGTAA |  | This study |
|  | kdgM2-F | CTTATCAGCACCAGCACAAT |  | This study |
|  | kdgM2-R | ACCGTTACTCACACCTTCA |  | This study |
|  | kdgR-F | TCGTGGTGAAGTAGAAGAGAT |  | This study |
|  | kdgR-R | CAGGCACAGCGATACAAC |  | This study |
|  | pelP-F | AAGTCAATATAGACACCACCAACAG |  | This study |
|  | pelP-R | TATGGCATCAACATCGGCATA |  | This study |

The table shows the sequences of primers used for cloning, the construction of translational and transcriptional fusions, and to generate fragments for protein-DNA binding assays (EMSAs).

*- 5’ extensions added to introduce cleavage sites for the indicated restriction enzymes **are shown in bold.**

**REFERENCES**

Nieckarz, M., Raczkowska, A., Dębski, J., Kistowski, M., Dadlez, M., Heesemann, J., et al. (2016). Impact of OmpR on the membrane proteome of Yersinia enterocolitica in different environments: repression of major adhesin YadA and heme receptor HemR. *Environ*. *Microbiol*. 18, 997–1021, doi: 10.1111/1462-2920.13165.

Wannet, W.J., Reessink, M., Brunings, H.A, and Maas, H.M. (2001). Detection of pathogenic *Yersinia enterocolitica* by a rapid and sensitive duplex PCR assay*. J. Clin. Microbiol*. 39, 4483–4486. doi: 10.1128/JCM.39.12.4483-4486.2001
