# Supplementary material for: The Role of OmpR in the Expression of Genes of the KdgR Regulon Involved in the Uptake and Depolymerization of Oligogalacturonides in Yersinia enterocolitica
Source: Front Cell Infect Microbiol. 2017 Aug 15;7:366. doi: 10.3389/fcimb.2017.00366 (PMC5559549; doi:10.3389/fcimb.2017.00366)
Supplement: Additional File 1 — Bioinformatic analysis of Y. enterocolitica proteins of the KdgM family. [file DataSheet1.docx]

**Bioinformatic analysis of *Y. enterocolitica* proteins of the KdgM family**

Bioinformatic analysis of the *Y. enterocolitica* subsp. *palearctica* 105.5R(r) (3/O:9 bio-serotype) genome (NCBI Reference Sequence: NC_015224.1) revealed the presence of two KdgM homologs: one encoded by the *kdgM1* gene (Acc. no. ADZ42456; nucleotide position 2,265,584 bp to 2,264,886 bp), with an ORF of 699 bp (232 aa protein including signal peptide), and the other by the *kdgM2* gene (Acc. no. ADZ44282; nucleotide position 4,365,460 bp to 4,364,756 bp), with an ORF of 705 bp (234 aa protein including signal peptide). The mature KdgM family porins of *Y. enterocolitica* subsp. *palearctica* 105.5R(r) are proteins of similar size: KdgM1 – 24,817 Da, KdgM2 – 24,724 Da.

Amino acid sequence alignment of KdgM1 and KdgM2 revealed 62% identity (**Supplementary Figure S1**). Both KdgM proteins of *Y. enterocolitica* exhibit 65% identity to KdgM and 57% identity to KdgN, the two oligogalacturonide-specific porins of *D. dadantii* (**Supplementary Figure S1**). Genes encoding KdgM1 and KdgM2 were identified in the genomes of another subspecies of *Y.* *enterocolitica*, subsp. *enterocolitica* 8081 (99% amino acid identity to KdgM1 and KdgM2 of subsp. *palearctica* 105.5R(r)) and also in other pathogenic *Yersiniae*, i.e. Y. pseudotuberculosis IP 32953 (KdgM1, 63% amino acid identity to KdgM and 54% amino acid identity to KdgN; KdgM2, 65% amino acid identity to KdgM and 53% amino acid identity to KdgN) and Y. pestis (CO92 and KIM10+) (KdgM1, 63% amino acid identity to KdgM and 54% amino acid identity to KdgN; KdgM2, 65% amino acid identity to KdgM and 53% amino acid identity to KdgN). Four KdgM homologs (KdgM, KdgN, KdgM3, KdgM4) present in the genome of *Pectobacterium carotovorum*, (formerly Erwinia carotovora; Rodionov et al., 2004) exhibit different levels of identity to *Y. enterocolitica* KdgM1 and KdgM2, with KdgM3 and KdgM4 being the least similar (**Supplementary Figure S1**).

**REFERENCES**

Rodionov, D. A., Gelfand, M. S., and Hugouvieux-Cotte-Pattat, N. (2004). Comparative genomics of the KdgR regulon in *Erwinia chrysanthemi* 3937 and other gamma-proteobacteria. *Microbiology* 150, 3571–3590. doi: 10.1099/mic.0.27041-0
